# On the interpretability of machine learning-based model for predicting hypertension

**DOI:** 10.1186/s12911-019-0874-0

**Published:** 2019-07-29

**Authors:** Radwa Elshawi, Mouaz H. Al-Mallah, Sherif Sakr

**Affiliations:** 1 Data Systems Group, Institute of Computer Science, University of Tartu, 2 J. Liivi St., 50409 Tartu, Estonia; 2 Houston Methodist Center, Tartu, Estonia

**Keywords:** Machine learning, Interpretability, Hypertension

## Abstract

**Background:**

Although complex machine learning models are commonly outperforming the traditional simple interpretable models, clinicians find it hard to understand and trust these complex models due to the lack of intuition and explanation of their predictions. The aim of this study to demonstrate the utility of various model-agnostic explanation techniques of machine learning models with a case study for analyzing the outcomes of the machine learning random forest model for predicting the individuals at risk of developing hypertension based on cardiorespiratory fitness data.

**Methods:**

The dataset used in this study contains information of 23,095 patients who underwent clinician-referred exercise treadmill stress testing at Henry Ford Health Systems between 1991 and 2009 and had a complete 10-year follow-up. Five *global* interpretability techniques (Feature Importance, Partial Dependence Plot, Individual Conditional Expectation, Feature Interaction, Global Surrogate Models) and two local interpretability techniques (Local Surrogate Models, Shapley Value) have been applied to present the role of the interpretability techniques on assisting the clinical staff to get better understanding and more trust of the outcomes of the machine learning-based predictions.

**Results:**

Several experiments have been conducted and reported. The results show that different interpretability techniques can shed light on different insights on the model behavior where global interpretations can enable clinicians to understand the entire conditional distribution modeled by the trained response function. In contrast, local interpretations promote the understanding of small parts of the conditional distribution for specific instances.

**Conclusions:**

Various interpretability techniques can vary in their explanations for the behavior of the machine learning model. The global interpretability techniques have the advantage that it can generalize over the entire population while local interpretability techniques focus on giving explanations at the level of instances. Both methods can be equally valid depending on the application need. Both methods are effective methods for assisting clinicians on the medical decision process, however, the clinicians will always remain to hold the final say on accepting or rejecting the outcome of the machine learning models and their explanations based on their domain expertise.

## Introduction

Machine learning prediction models have been used in different areas such as financial systems, advertising, marketing, criminal justice system, and medicine. The inability of machine learning users to interpret the outcomes of the complex machine learning models becomes problematic [1]. Machine learning *interpretability* is defined as the degree to which a machine learning user can understand and interpret the prediction made by a machine learning model [2, 3]. Despite the growing use of machine learning-based prediction models in the medical domains [4–7], clinicians still find it hard to rely on these models in practice for different reasons. First, most of the available predictive models target particular diseases and depend on domain knowledge of clinicians [8–10]. Applying such predictive models on large health information systems may not perform well because of the availability of multiple, complex data sources and the heterogeneous mixture of patients and diagnoses. Second, most of the models developed by data scientists mainly focus on prediction accuracy as a performance metric but rarely explain their prediction in a meaningful way [11, 12]. This is especially true with complex machine learning, commonly described as *black-box* models, such as Support Vector Machines [13], Random Forest [14] and Neural Networks [15].

Although many predictive models have been developed to predict the risk of hypertension [16–18], the frameworks for establishing trust and confidence for these predictions have been always missing. Thus, there has been some criticism for using machine learning models in the medical domain even with their promise of high accuracy [19]. In practice, addressing this issue is critical for different reasons, especially if clinicians are expected to use these models in practice. First, explaining the predictions of the developed model contributes to the trust problem by enabling clinicians to make sure that the model makes the right predictions for the right reasons and wrong predictions for the right reasons. Second, explaining predictions is always useful for getting some insights into how this model is working and helps in improving model performance. Since May 2018, the General Data Protection Regulation (GDPR) forces industries to explain any decision taken by a machine when automated decision making takes place: “*a right of explanation for all individuals to obtain meaningful explanations of the logic involved*”, and thus increases the efforts of developing interpretable and explainable prediction models [20].

In our previous study [21], we evaluated the performance of several machine learning techniques on predicting individuals at risk of developing hypertension using cardiorespiratory fitness data. In particular, we evaluated and compared six well-known machine learning techniques: *LogitBoost*, *Bayesian Network*, *Locally Weighted Naive Bayes*, *Artificial Neural Network*, *Support Vector Machine,* and *Random Forest*. Using different validation methods, the Random Forest model, a complex ensembling machine learning model, has shown the maximum area under the curve (AUC = 0.93). The attributes used in in the *Random Forest* model are *Age, METS, Resting Systolic Blood Pressure, Peak Diastolic Blood Pressure, Resting Diastolic Blood Pressure, HX Coronary Artery Disease, Reason for test, History of Diabetes, Percentage HR achieved, Race, History of Hyperlipidemia, Aspirin Use, Hypertension response*. In this study, we apply various techniques to present complete interpretation for the best performing model (Random Forest) in predicting individuals at risk of developing hypertension in an understandable manner for clinicians either at the global level of the model or the local level of specific instances. We believe that this study is an important step on improving the understanding and trust of intelligible healthcare analytics through inducting a comprehensive set of explanations for prediction of local and global levels. The remainder of this paper is organized as follows. In Section 2, we highlight the main interpretability techniques considered in this work. Related work is discussed in Section 3. In Section 4, we introduce the dataset employed in our experiments and discuss the interpretability methodologies. Results are presented in Section 5. In Section 6, we discuss our results. Threats to the validity of this study are discussed in Section 7 before we finally draw the main conclusions in Section 8.

## Background

One simple question that can be posed is “*Why we do not simply use interpretable models, white-box models, such as linear regression or decision tree?*”. For example, linear models [22] present the relationship between the independent variables (input) and the target (output) variable as a linear relationship that is commonly described by weighted equations which makes the prediction procedure a straightforward process. Thus, linear models and decision tree have broad usage in different domains such as medicine, sociology, psychology, and various quantitative research fields [23–25]. The decision tree [26] is another example where the dataset is split based on particular cutoff values and conditions in a tree shape where each record in the dataset belongs to only one subset, leaf node. In decision trees, predicting the outcome of an instance is done by navigating the tree from the root node of the tree down to a leaf and thus the interpretation of the prediction is pretty straightforward using a nice natural visualization. However, in practice, even though *black-box* models such as Neural Networks can achieve better performance than *white-box* models (e.g. linear regression, decision tree), they are less interpretable.

In general, methods for machine learning interpretability can be classified as either *Model-Specific* or *Model-Agnostic*. In principle, model-specific interpretation methods are limited to specific types of models. For example, the interpretation of regression weights in a linear model is a model-specific interpretation and does not work for any other model. On the other hand, model-agnostic interpretation methods are more general, can be applied on any machine learning model and are usually post hoc [27]. This facilitates the comparison of different types of interpretability techniques and eliminates the need to replace the interpretability technique when the models are replaced, so such techniques are more flexible and usable [27]. These agnostic techniques work by analyzing pairs of input features and output without depending on the underlying model. Model-Agnostic techniques also have some challenges [27]. One challenge is that it is hard to get a global understanding of complex models due to the trade-off between model interpretability and model flexibility. In some applications, an exact explanation may be a must and using such black-box techniques is not accepted. In this case, using an interpretable model such as a linear regression model is preferable and the same holds for any application in which interpretability is more important than model performance. Another challenge is to make model-agnostic explanations actionable. It is easier to incorporate user feedback into the model implemented using explainable models rather than using a black-box model [28].

Another way to classify machine learning interpretability methods is based on whether the interpretation of the model is *global* or *local*. In principle, global interpretations enable a clinician to understand the entire conditional distribution modeled by the trained response function. They are obtained based on average values. In contrast, local interpretations promote the understanding of small parts of the conditional distribution. Since conditional distribution decomposes of small parts that are more likely to be linear or well-behaved and hence can be explained by interpretable models such as linear regression and decision trees.

In this study, we apply various *global* and *local model-agnostic* methods that facilitate global model interpretation and local instance interpretation of a model that has been used in our previous study [21]. In particular, in our previous study, we evaluated and compared the performance of six machine learning models on predicting the risk of hypertension using cardiorespiratory fitness data of 23,095 patients who underwent treadmill stress testing at Henry Ford Health hospitals over the period between 1991 and 2009 and had a complte10-year follow-up. The six machine learning models evaluated were logit boost, Bayesian network, locally weighted naive Bayes, artificial neural network, support vector machine and random forest. Among such models, random forest achieved the highest performance of AUC = 0.93.

**Fig. 1:**
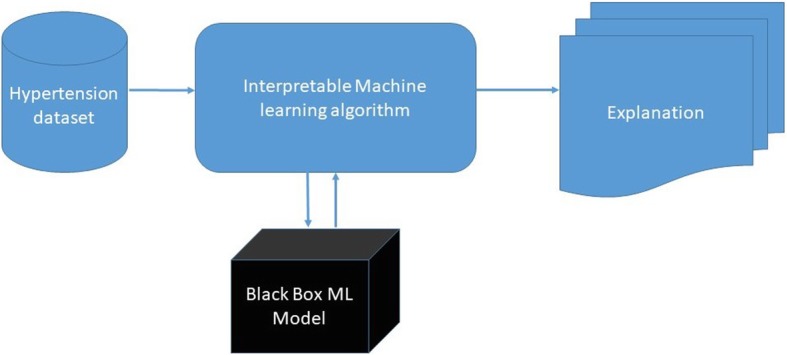
The interpretability process of black box machine learning algorithms

Figure 1 illustrates the steps of our interpretation process.

## Related work

The volume of research in machine learning interpretability is growing rapidly over the last few years. One way to explain complex machine models is to use interpretable models such as linear models and decision trees to explain the behavior of complex models. LIME interpretability technique explains the prediction of complex machine model by fitting an interpretable model on perturbed data in the neighborhood of the instance to be explained. Decision trees have been used intensively as a proxy model to explain complex models. Decision trees have several desirable properties [29]. Firstly, due to its graphical presentation, it allows users to easily have an overview of complex models. Secondly, the most important features that affect the model prediction are shown further to the top of the tree, which show the relative importance of features in the prediction. Lots of work consider decomposing neural networks into decision trees with the main focus on shallow networks [30, 31].

Decision rules have used intensively to mimic the behavior of a black-box model globally or locally given that the training data is available when providing local explanations [32]. Koh and Liang [33] used influence functions to find the most influential training examples that lead to a particular decision. This method requires access to the training dataset used in training the black-box model. Anchors [34] is an extension of LIME that uses a bandit algorithm to generate decision rules with high precision and coverage. Another notable rule-extraction technique is MofN algorithm [35], which tries to extract rules that explain single neurons by clustering and ignoring the least significant neurons. The FERNN algorithm [36] is another interpretability technique that uses a decision tree and identifies the meaningful hidden neurons and inputs to a particular network.

Another common interpretability technique is saliency maps that aim to explain neural networks models by identifying the significance of individual outcomes as an overlay on the original input [37]. Saliency-based interpretability techniques are popular means for visualizing the of a large number of features such as images and text data. Saliency maps can be computed efficiently when neural network parameters can be inspected by computing the input gradient [38]. Derivatives may miss some essential aspects of information that flows through the network being explained and hence some other approaches have considered propagating quantities other than gradient through the network [39–41].

Interpretability of black-box models via visualization has been used extensively [42–44]. Several tools have been designed to provide an explanation for the importance of features for random forest predictions [45], however, these tools are model-specific and cannot be generalized to other models. The authors of [46, 47] discussed several methods for extracting rules from neural networks. Poulet [48] presented a methodology for explaining the prediction model by assigned a contribution value for each feature using visualization technique. However, this work has been only able to handle linear additive models. Strumbelj et al. [49] provided insights for explaining the predictions of breast cancer recurrence by assigning a contribution value to each feature, which could be positive, negative, or zero. A positive contribution means that the feature supports the prediction of the class of interest, a negative contribution means that the feature is against the prediction of the class of interest, and zero means that the feature has no influence on the prediction of the class of interest. Caruana et al. [50] presented an explanation technique which is based on selecting the most similar instances in the training dataset to the instance to be explained. This type of explanation is called case-based explanation and uses the k-nearest neighbors (KNN) algorithm to find the k nearest examples close to the instance to be explained based on a particular distance metric such as Euclidean distance [51].

## Research design and methods

In this section, we describe the charchteristics of the cohort of our study. In addition, we describe the global and local intepretability techniques which we used for explaining the predictions of the model that has been developed for predicting the risk of hypertension using cardiorespiratory fitness data.

### Cohort study

The dataset of this study has been collected from patients who underwent treadmill stress testing by physician referrals at Henry Ford Affiliated Hospitals in metropolitan Detroit, MI in the U.S. The data has been obtained from the electronic medical records, administrative databases, and the linked claim files and death registry of the hospital [52]. Study participants underwent routine clinical treadmill exercise stress testing using the standard Bruce protocol between January 1st, 1991 and May 28th, 2009. The total number of patients included in this study is (*n* = 23,095). The data set includes 43 attributes containing information on vital signs, diagnosis and clinical laboratory measurements. The baseline characteristics of the included cohort are shown in Table 1. The dataset contains 23,095 individuals (12,694 males (55%) and 10,401 (45%) females) with ages that range between 17 and 96. Half of the patients have a family history of cardiovascular diseases. During the 10-years follow-up, around 35% of the patients experienced hypertension. Male hypertension patients represent around 55% of the total hypertension patients while female patients represent around 44% of the total hypertension patients. For more details about the dataset, the process of developing the prediction model and the FIT project, we refer the reader to [21, 52].

**Table 1:** Dataset Description (Cohort Characteristics)

Age	49+/− 12
Gender	
Male	12,694 (55%)
Female	10,401 (45%)
Race	
Black	4694 (20%)
Other	18401 (80%)
Reason for Test	
Chest Pain	12581 (54%)
Shortness of Breath	1956 (8%)
Pre-Operation	255 (1%)
Known Coronary Artery Disease	524 (2%)
Rule out Ischemia	2286 (10%)
Abnormal prior test	1004 (4%)
Stress	
Peak METS (Mean +/− SD)	10.2 +/− 2.79
Resting Systolic Blood Pressure (Mean +/− SD)	124 +/− 17
Resting Diastolic Blood Pressure (Mean +/− SD)	79 +/− 10
Resting Heart rate (Mean +/− SD) beat per minute (bpm)	73 +/− 12
Peak Diastolic Blood Pressure (Mean +/− SD)	82 +/− 13
Peak Heart Rate (Mean +/− SD) beat per minute (bpm)	159 +/− 17
Past Medical History	
Diabetes	1887 (8%)
History of Smoking	9,518 (41%)
Family History	11,865 (51%)
History of Hyperlipidemia	7,769 (34%)
History of Coronary Artery Bypass Graft	314 (1%)

In the following, we highlight the interpretability methods that are used in this study.

### Global interpretability techniques

Table 2 summarizes the main features of the model-agnostic interpretability techniques used in this study. In the following, we list and explain each of them.

**Table 2:** Main features of the model-agnostic interpretability techniques used in this study

Technique	Global	Local	Advantages	Disadvantages
Feature Importance	✓		• Highly compressed global interpretation • Consider interactions between features	Unclear whether it can be used on training dataset or testing dataset
Partial Dependence Plot	✓		Intuitive and clear interpretation	Assumption of independence between features
Individual Conditional Expectation	✓		Intuitive and easy to understand	Plot can become overcrowded to understand
Feature Interaction	✓		Detects all interactions been features	Computationally expensive
Global Surrogate Models	✓		Easy to measure the goodness of your surrogate model using R-squared measure	Not clear what is the best cut-off for R-squared to trust the resulted surrogate model
Local Surrogate Model (LIME)		✓	• Short and comprehensible explanation. • Explains different types of data (tabular, text and image)	• Instability of the explanation • Very close points may have totally different explanations
Shapley Value Explanations		✓	Explanation is based on strong game theory theorem	Computationally very expensive

#### Feature Importance

It is a global interpretation method where the feature importance is defined as the increase in the model’s prediction error after we permuted the values of the features (breaks the relationship between the feature and the outcome) [53]. A feature is considered important if permuting its values increase the error (degrade the performance).

#### Partial Dependence Plot (PDP)

It is a global interpretation method where the plot shows the marginal effect of a single feature on the predicted risk of hypertension of a previously fit model [54]. The prediction function is fixed at a few values of the chosen features and averaged over the other features. Partial dependence plots are interpreted in the same way of a regression model which makes its interpretation easy. The main disadvantage of the partial dependence plot is the assumption that the feature of which the PDP is computed to be completely independent distributed from the other features that we average over.

#### Individual Conditional Expectation (ICE)

The partial dependence plot aims to visualize the average effect of a feature on the predicted risk of hypertension. Partial dependence is a global method as it does not focus on specific instances but on an overall average. ICE plot can be seen as the disaggregated view of PDP by displaying the estimated functional relationship for each instance in the dataset. The partial dependence plot can be seen as the average of the lines of an ICE plot [55]. In other words, ICE visualizes the dependence of the predicted risk of hypertension on particular features for each instance in the dataset. One main advantage of the ICE is that is easier to understand and more intuitive to interpret than the PDP. ICE suffers from the same disadvantage of PDP.

#### Feature Interaction

It is a global interpretation method where the interaction between two features represents the change in the prediction that occurs by varying the 13 features, after having accounted for the individual feature effects. It presents the effect that comes on top of the sum of the individual feature effects. One way to measure the interaction strength is to measure how much of the variation of the predicted outcome depends on the interaction of the features. This measure is known as H-statistic [56]. One of the main advantages of the feature interaction is that it considers the interaction between the features. The main disadvantage of the feature interaction is that it is computationally expensive as it iterates over all the instances in the dataset.

#### Global Surrogate Models

It is a global interpretation method which aims to approximate the predictions of a complex machine learning models (such as neural networks) using a simple interpretable machine learning models (such as linear regression) [57]. Global surrogate models are considered model-agnostic methods as they do not require any information about the internal workings and the hyper-parameters settings of the black-box model. One way to obtain a surrogate model is as follows. Train an interpretable model such as logistic regression or decision tree on the same dataset used to train the black-box model (or a dataset that has the same distribution) such that target for the interpretable model is the predictions of the black-box model. The main advantage of the surrogate models is its flexibility, in addition, it is easy to assess how well it approximates the black-box model. However, it is still problematic how well the surrogate model should approximate the black-box model in order to be trusted.

### Local interpretability techniques

#### Local Surrogate Models (LIME)

It is a local model agnostic interpretation method which focuses on explaining the prediction of a single prediction of any black-box machine learning model locally (within the neighborhood of the prediction instance to be explained) [58]. The idea of LIME is quite intuitive, it generates a new dataset that consists of perturbed samples and then gets the associated predictions from the black box model. Next, LIME weight perturbed samples by how close they are from the point to be explained where the closer the point form the point to be explained, the higher weight it takes. Then, LIME fits an interpretable model (such as linear regression) on the weighted sampled instances. The learned model should be a good approximation of the machine learning model locally, but not globally.

#### Shapley Value Explanations

It is a local interpretation method from game theory [59]. This interpretation method assumes that each feature in the instance to be explained is a ‘player’ in a game and the prediction is the payout. The Shapley value aims to distribute the payout among the features in a fair way. The main idea of Shapley value is that for each feature *f* in the instance to be explained, evaluate the model using all possible coalitions (sets) of features with and without *f*. Such approach is extremely computationally expensive as the number of the coalitions increases exponentially with the number of features. Strumbelj and Kononenko [57], presented an approximation algorithm for Shapley Values using Monte-Carlo sampling technique. This approximation algorithm has been used in this work as an example of local explainer and will be referred to as Shapley Values explainer.

The analysis of the global and local machine learning interpretability techniques has been conducted using R-based ML packages (Version 3.3.1) (https://www.r-project.org/).

## Results

In this section we present the results of applying various gloal and local interpretability techniques for our predictive model for the individuals at risk of developing hypertension based on cardiorespiratory fitness data. In particular, we present the results of Five global interpretability techniques, namely, feature importance, partial dependence plot, individual conditional expectation, feature interaction and global surrogate models. In addition, we present the results of 2 local explanation techniques, namely, LIME and Shapley value explanation.

### Global interpretability techniques

#### Feature Importance

**Fig. 2:**
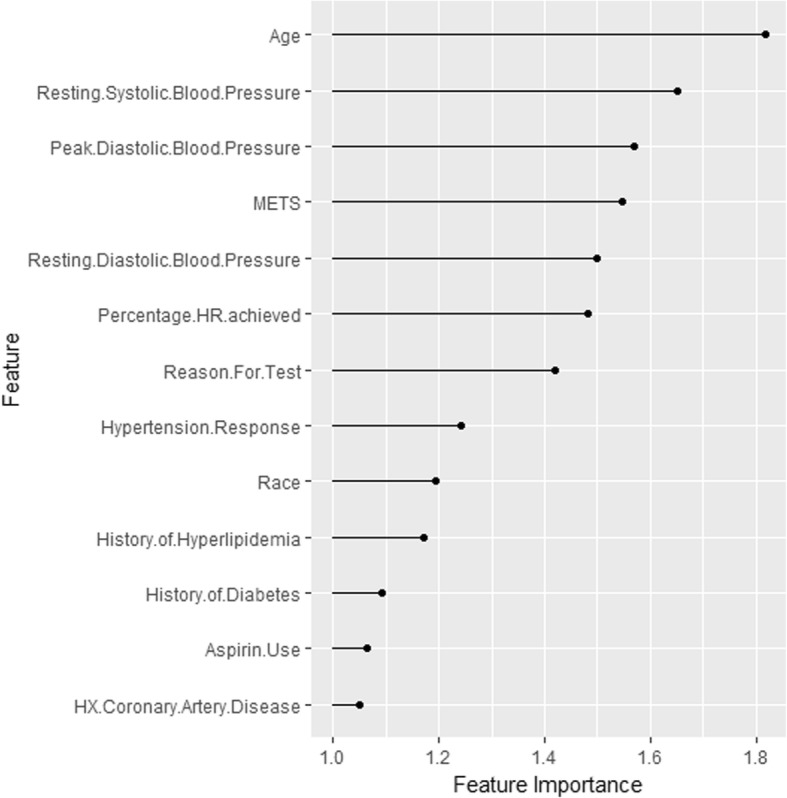
The importance for each feature in predicting the high risk of hypertension

Figure 2 shows the ranking of the importance of the selected input features in predicting the high risk of hypertension. The feature importance represents the factor by which the error is increased compared to the original model error. As shown in the figure, *Age* is the most important feature, followed by *Resting Systolic Blood Pressure*. The *History of Coronary Artery Disease* is the least significant feature.

#### Partial Dependence Plot and Individual conditional expectation plot

**Fig. 3:**
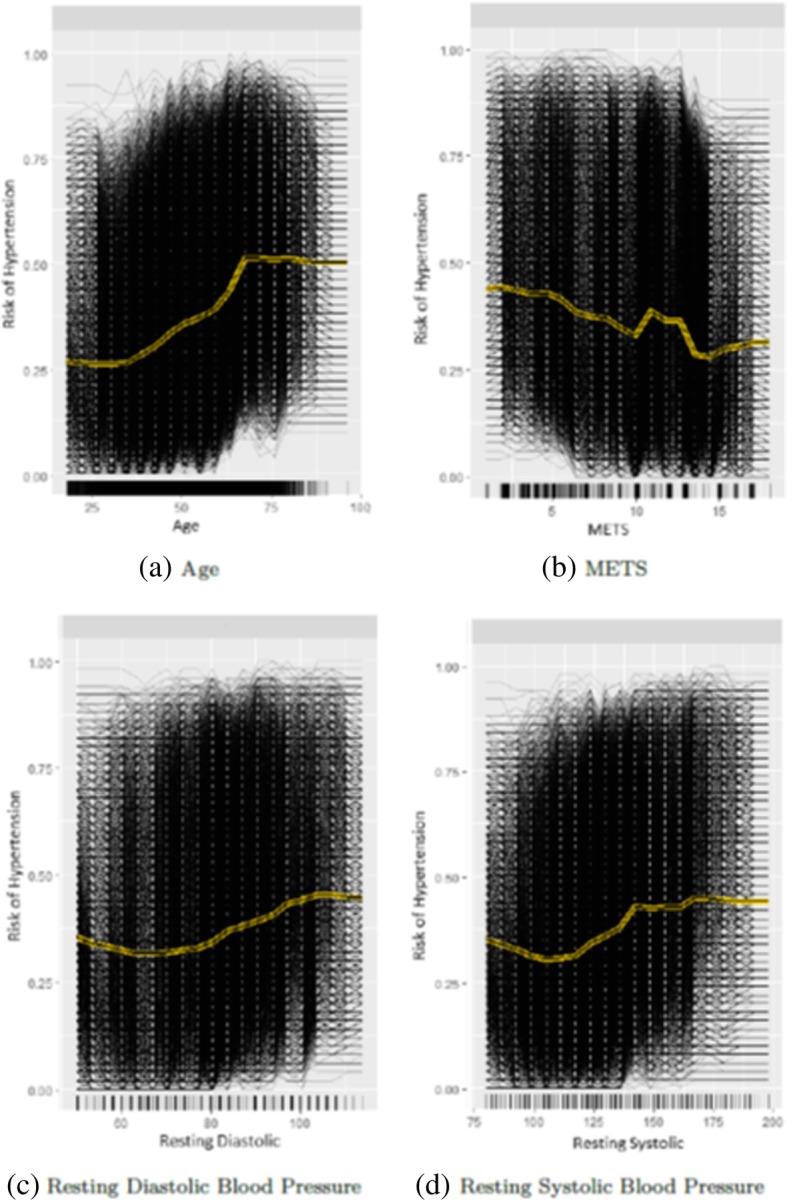
Partial dependence plots for the highly ranked features for predicting hypertension

The yellow line in Fig. 3 shows the partial dependence plot of the probability of high risk of hypertension for each of the highly ranked features for predicting hypertension: *Age*, *METS*, *Resting Systolic Blood Pressure* and *Resting Diastolic Blood Pressure*. The black lines in Fig. 3 show the individual conditional expectation plot of the high risk of hypertension probability of the features. Each of the black lines represents the conditional expectation for one patient. For the *Age* feature, the partial dependence plot shows that, on average, the probability of high risk of hypertension increases gradually from 0.25 to reach 0.5 at the age of 65 and then remain stable till the age of 100 (Fig. 3a). For the *METS* feature, the partial dependence plot shows that, on average, the increase in *METS* is associated with a lower probability of high risk of hypertension (Fig. 3b). On average, the increase in the *Resting Diastolic Blood Pressure* is associated with a gradual increase in the probability of high risk of hypertension (Fig. 3c). For the *Resting Systolic Blood Pressure*, the plot shows that the probability of high risk of hypertension increases from 0.30 to 0.40 at METS around 140, then slightly fluctuating around 0.40 (Fig. 3d).

#### Feature Interaction

**Fig. 4:**
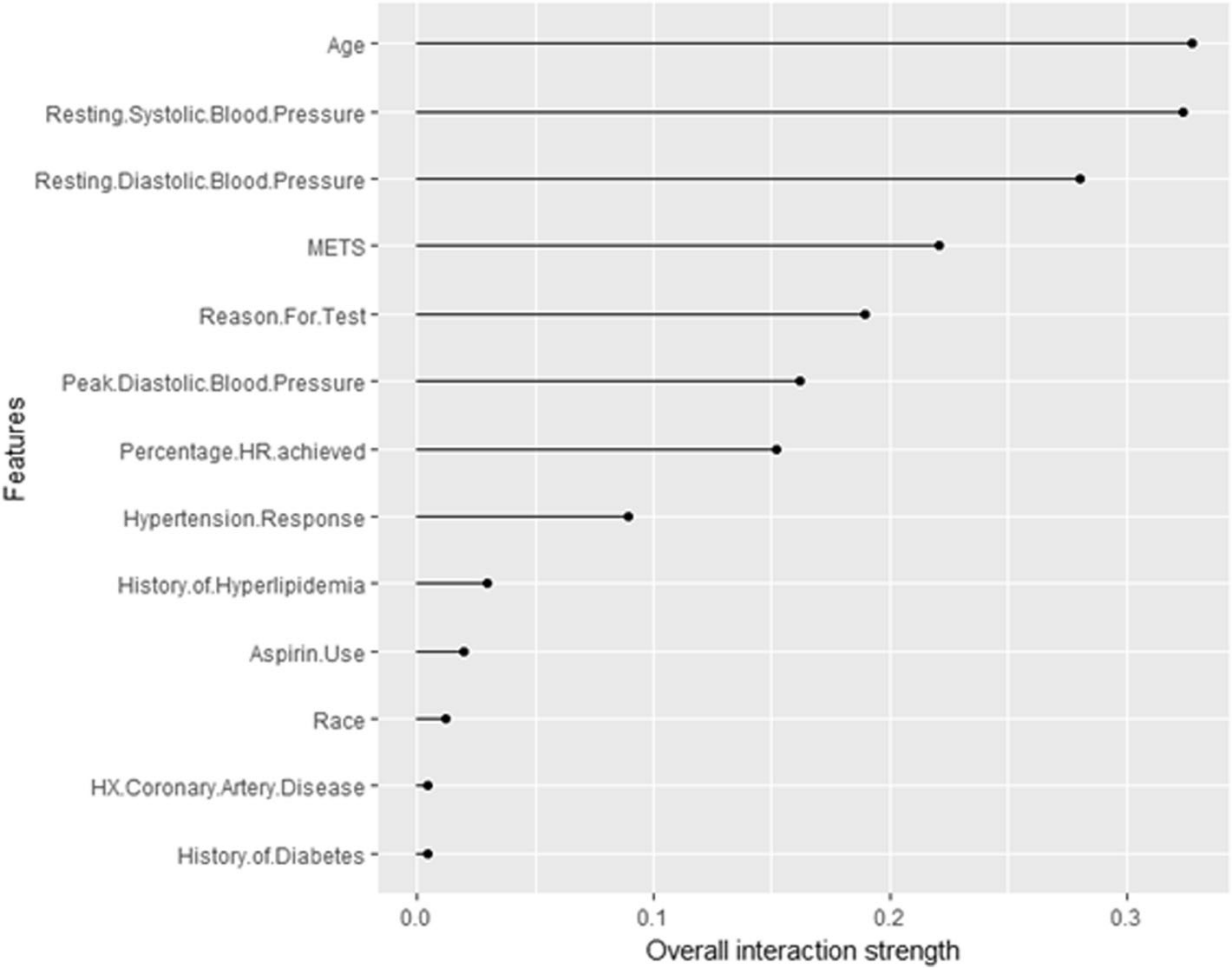
The interaction strength for each of the input features with all other features for predicting the high risk of hypertension

Figure 4 shows the interaction strength for each of the input features with all other features for predicting the probability of high risk of hypertension. The *Age* has the highest interaction effect with all other features, followed by the *Resting Systolic Blood Pressure*. The *History of Diabetes* has the least interaction with all other features. Overall the interaction effects between the features are considerably strong.

#### Global Surrogate Models

**Fig. 5:**
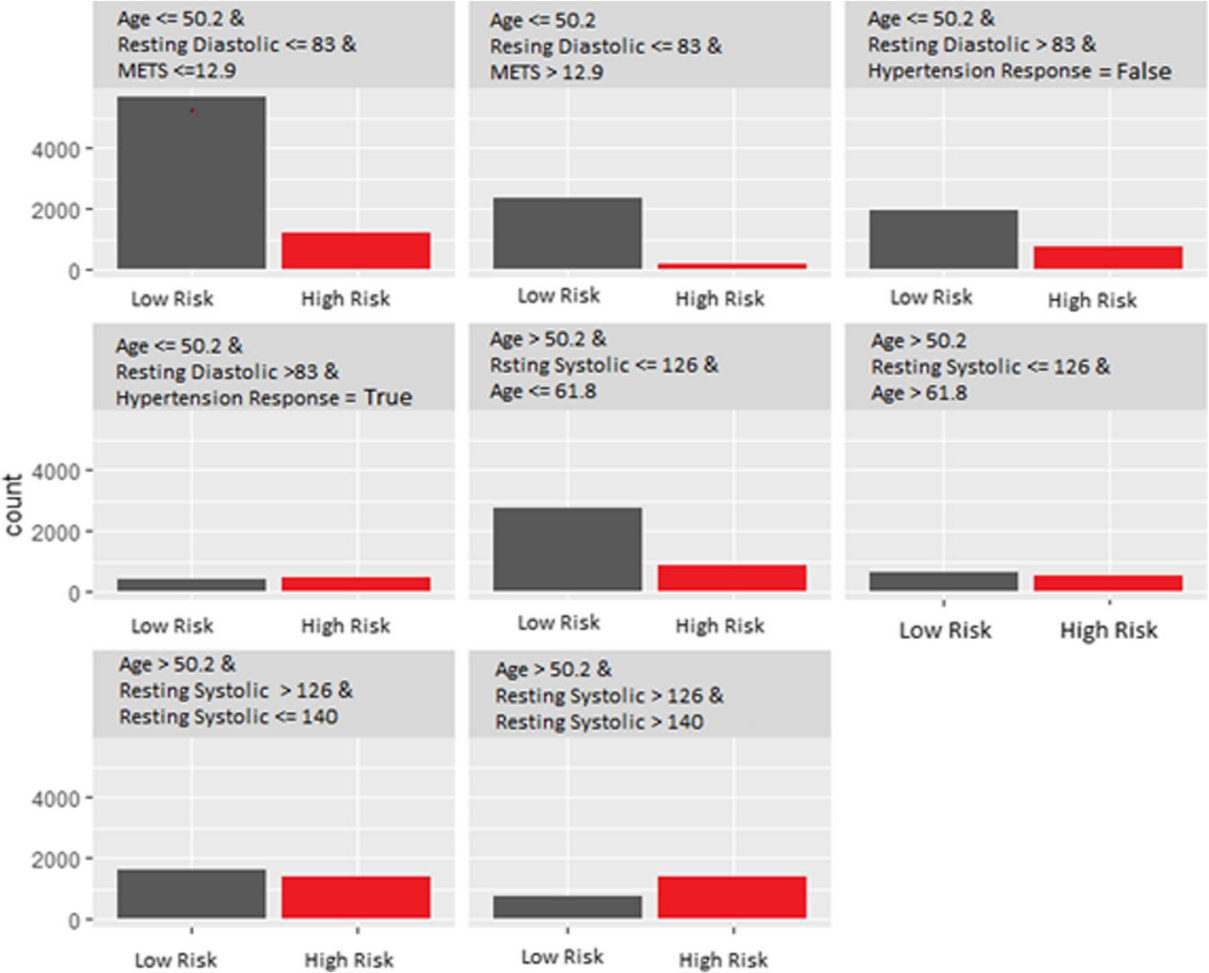
The terminal nodes of a surrogate tree of depth equals to 3 that approximates the behavior of the black box random forest model trained on the hypertension dataset

**Fig. 6:**
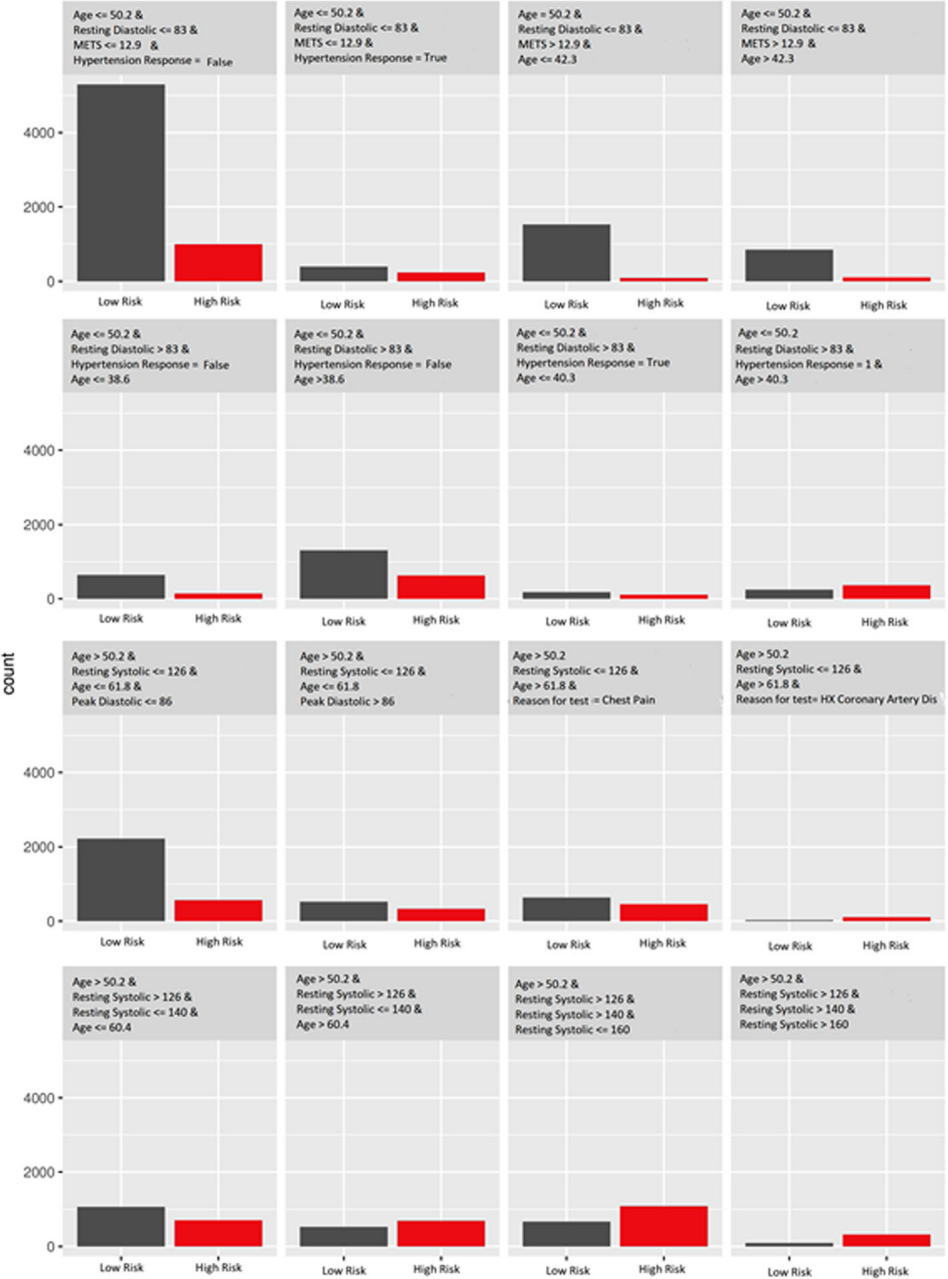
The terminal nodes of a surrogate tree of depth equals to 4 that approximates the behavior of the black box random forest model trained on the hypertension dataset

We fit a decision tree of depths equal to 3 and 4, using the original dataset, but with the prediction of the model (Random Forest) used as an outcome for the decision tree model, instead of the real classes (high risk of hypertension and low risk of hypertension) from the original dataset. Figures 5 and 6 show the terminal nodes of a surrogate decision tree of depth equals to 3 and 4 respectively. The counts in the nodes show the distribution of the random forest model predictions in the nodes. The counts in the nodes in Fig. 5 show that the surrogate tree predicts a higher number of low risk of hypertension patients when the *Age* is less than or equal to 50:2, *Resting Diastolic Blood Pressure* is less than or equal to 83 and *METS* is less than or equal to 12:9. Also, the counts show that the surrogate tree of depth 3 predicts a higher number of high risk of hypertension patients when the *Age* is greater than 50:2, *Resting Systolic Blood Pressure* is between 126 and 140. One way to measure how well the surrogate replicates the black box model is the R-squared measure. The surrogate tree of depth 3 has an R-squared (variance explained) around 0:3 which means that the tree model of depth 3 approximates the underlying Random Forest behavior very poorly. The counts of the nodes in Fig. 6 show that the surrogate tree of depth 4 predicts a higher number of low risk of hypertension patients when the *Age* is less than or equal to 50.2, *Resting Diastolic Blood Pressure* is less than or equal to 83, *METS* is less than or equal to 12.9 and Hypertension Response is false. The counts in Fig. 6 also shows that the surrogate model predicts a higher number of high risk of hypertension patients when the *Age* greater than 50.2, *Resting Systolic Blood Pressure* is between 140 and 160. The R-squared of the surrogate model of depth 4 increases slightly to 0.4, however, when compared to the surrogate tree of depth 3, the model still does not approximate the black-box model (Random Forest) well.

### Local interpretability techniques

The explanatory plot produced by the LIME explanation mechanism illustrates for each feature and class, in which the range of values of a representative data point would fall. If it does, this gets counted as support for this prediction and if it does not, it gets scored as contradictory. In addition, LIME produces what is so-called *Explanation fit* that refers to the R-squared of the linear Ridge regression model which is fitted locally to explain the variance in the neighborhood of the examined instance. The explanatory plot produced by the Shapley Values explainer is close to the one generated by LIME in the sense that it shows the features’ names and features’ contributions that are used in the explanation. A feature with a positive contribution value means that the feature contributes toward increasing the prediction of the model and a feature with a negative value means that the feature contributing toward decreasing the model’s output. The sum of all features’ contributions is the difference between the black-box model output and the model’s output when no information is given about features’ values. Therefore, we can measure the change in the model’s output and hence identify the features that contribute to this change and the amount of each feature-value’s influence.

Since LIME and Shapley Values explainers are instance based explainers, in the following we evaluate both explainers based on 20 randomly selected instances from the testing dataset. In the following, we present the explanation of 20 instances in detail. We present 2 instances that have been correctly predicted by the black-box prediction model, one instance from the *True Positive* (correctly predicted as high risk of hypertension) group and another instance for the *True Negative* (correctly predicted as low risk of hypertension) group. In general, the generated explanations for the correctly predicted instances are commonly very intuitive and clear. They mostly follow common standard patterns. Thus, we chose to more focus on the incorrectly predicted instances as understanding the rationale and explanations for such incorrect predictions of the model increases the trust of the clinicians on the model behavior and performance. Thus, we present instances that comprehensively cover the *False Positive* and *False Negative* groups with consideration of the most important prediction factor, the patient’s age.

#### Instance 1 (True negative)

**Fig. 7:**
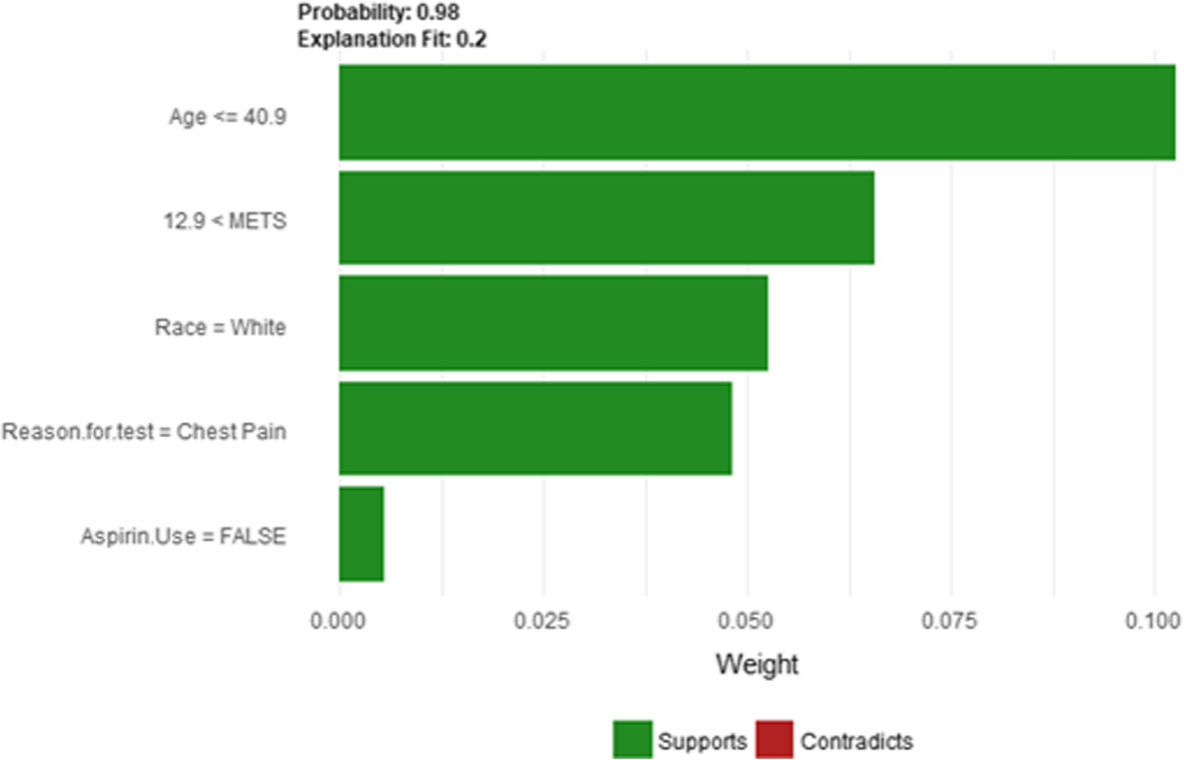
LIME explanation for Instance 1 as True Negative

The description of this instance is as follows: *Age = 36, METS = 13, Resting Systolic Blood Pressure = 80, Peak Diastolic Blood Pressure = 70, Resting Diastolic Blood Pressure = 60, HX Coronary Artery Disease = false, Reason for test = chest pain, HX Diabetes = false, Percentage HR achieved = 0.98, Race = white, Hx Hyperlipidemia = false, Aspirin Use = false, Hypertension Response = false.* Figure 7 *shows LIME explanation of the prediction of instance 1 as low risk of hypertension with a strong probability of 0:98. The explanation is created based on five features Age, METS, Race, Reason for test and Aspirin Use.*

**Fig. 8:**
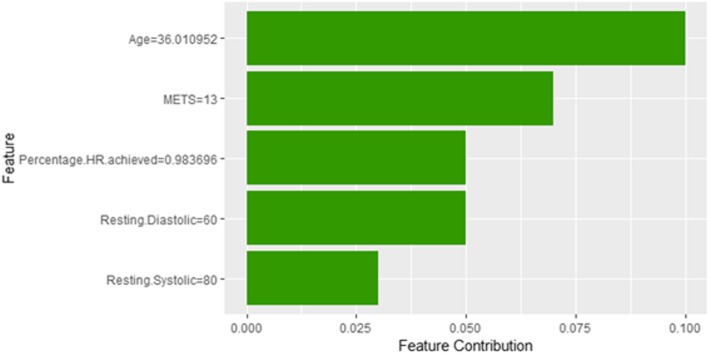
Shapley explanation for Instance 1 as True Negative

Figure 8 shows Shapley explanation of instance 1 based on five features *Age*, METS, *Percentage HR achieved*, *Resting Diastolic Blood Pressure* and *Resting Systolic Blood Pressure*. The *Age*, *METS* are the most important features that contributed to the prediction of low risk of hypertension for both LIME and Shapley. The explanations show that young patients under the age of 40s are at lower risk of developing hypertension compared to people above 40s which matches the partial dependence plot created in Fig. 3a and comes inline with the medical study by Rockwood et al. [60]. The explanations also show that those people whose METS are greater than 12:9 are at low risk of developing hypertension which matches the medical study by Juraschek et al. [61]. LIME explanation also shows that white people are at lower risk of developing hypertension compared to black people which is supported by the study conducted by Ergul et al. [62].

#### Instance 2 (True Positive)

The description of this instance is as follows: *Age = 64.8, METS = 7, Resting Systolic Blood Pressure = 110, Peak Diastolic Blood Pressure = 90, Resting Diastolic Blood Pressure = 70, HX Coronary Artery Disease = True, Reason for test = HX Coronary Artery Disease, HX Diabetes = false, Percentage HR achieved = 0.79, Race = black, Hx Hyperlipidemia = false, Aspirin Use = false, Hypertension Response = False.*

**Fig. 9:**
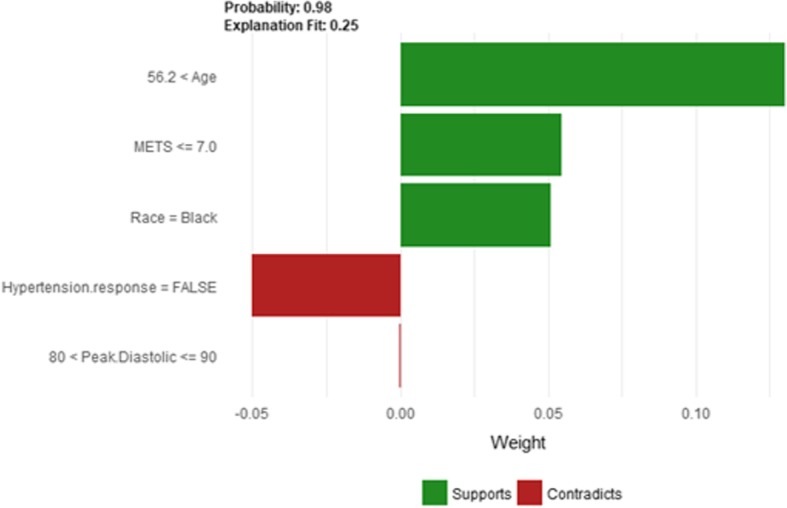
LIME explanation for Instance 2 as True Positive

**Fig. 10:**
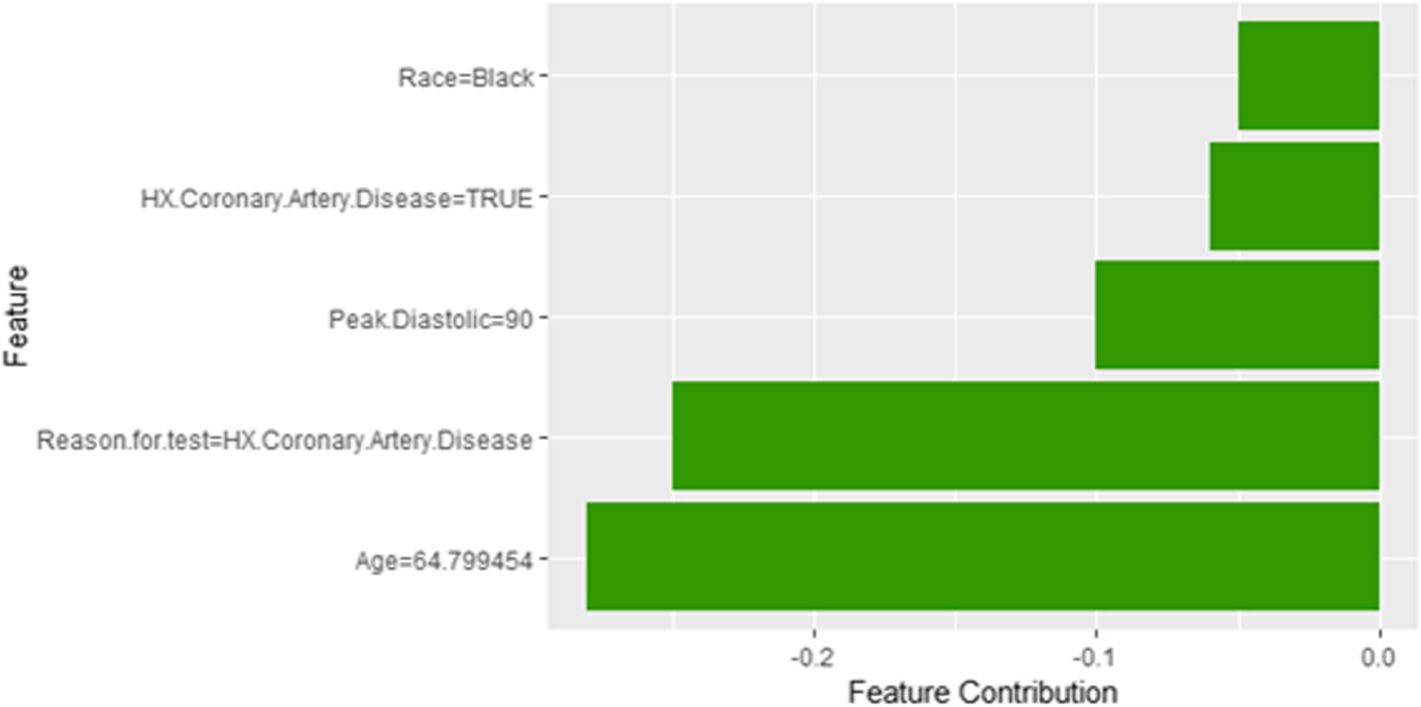
Shapley explanation for Instance 2 as True Positive

Figure 9 shows the LIME explanation of the prediction of the black-box model for instance 2 as high risk of hypertension (assigning a strong probability of 0.98 for high risk of hypertension). The explanation is created based on five features *Age*, *METS*, *Race*, *Hypertension Response*, and *Peak Diastolic Blood Pressure*. The three features *Age*, *METS,* and Race positively support the explanation as a high risk of hypertension. Having negative Hypertension Response test negatively contributed to the explanation of the high risk of hypertension which is inline with the medical study by Zanettini et al. [63]. Figure 10 shows the Shapley Values explanation of instance 2 as high risk of hypertension. The explanation is based on five features *Race*, *HX Coronary Artery Disease*, *Peak Diastolic Blood Pressure*, *Reason for test* and *Age* that all contribute toward decreasing of the probability of high risk of hypertension.

In the following, we are going to have a deep look at the misclassified instances by the Random Forest model and see the explanation using LIME. To ensure diversity, we selected nine instances from each of the *False Positive* instances (incorrectly classified as high risk of hypertension) and *False Negative* instances (incorrectly classified as low risk of hypertension) based on the patient’s age as it has been identified to be the most important feature based on the feature importance plot and the partial dependence plot.

**Fig. 11:**
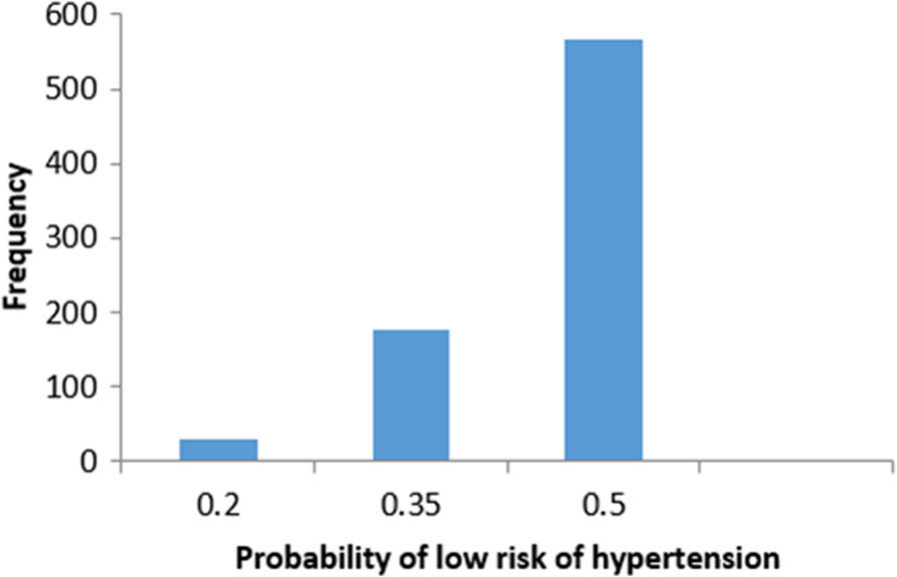
Histogram of false positive instances

We start studying false positive instances. Figure 11 shows the frequency distribution of the false positive instances based on the probability of low risk of hypertension. The probability of low risk of hypertension has been split into three groups (bins). **Group 1** represents instances with the probability of low risk of hypertension between [0–0.2]. **Group 2** and **Group 3** represent instances with the probability of low risk of hypertension that belongs to]0.2–0.35] and]0.35–0.5[, respectively. The frequency of the instances in group three is the highest (the black-box model predicts a patient as low risk of hypertension if the low-risk probability is greater than or equal to 0.5). In the following, we present sample instances from each of the three groups selected based on the patient’s age.

In the following, we present sample instances of *False Positive* predictions from **Group 1**. The instances are selected based on the patient’s age: one instance is close to the maximum age, one instance is close to the minimum age and one instance close to average age.

#### Instance 3 (False Positive Prediction of High Risk - Group 1 - Close to Maximum Age)

The description of this instance is as follows: *Age = 75.39, METS = 6.4, Resting Systolic Blood Pressure = 150, Peak Diastolic Blood Pressure = 90, Resting Diastolic Blood Pressure = 94, HX Coronary Artery Disease = false, Reason for test = HX Coronary Artery Disease, HX Diabetes = false, Percentage HR achieved = 1.04, Race = white, Hx Hyperlipidemia = true, Aspirin Use = true, Hypertension Response = true*.

**Fig. 12:**
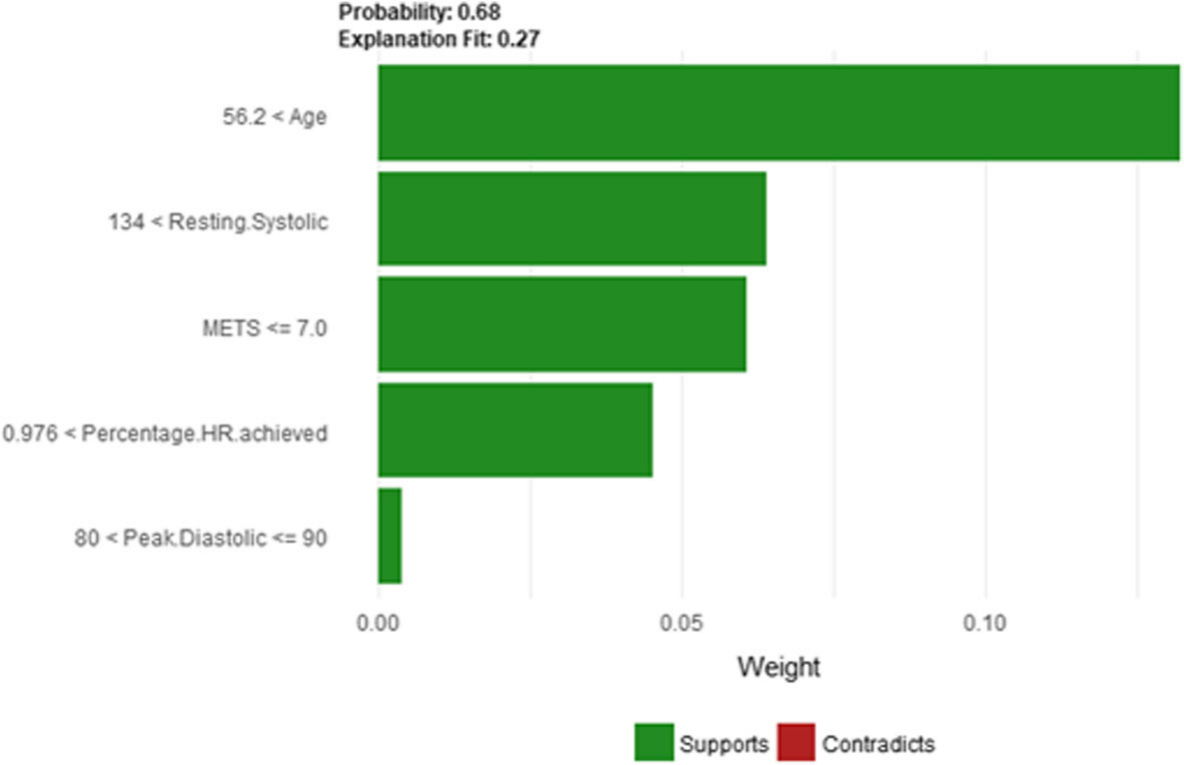
LIME explanation of Instance 3 as False Positive Prediction of High Risk - Group 1 - Close to Maximum Age

**Fig. 13:**
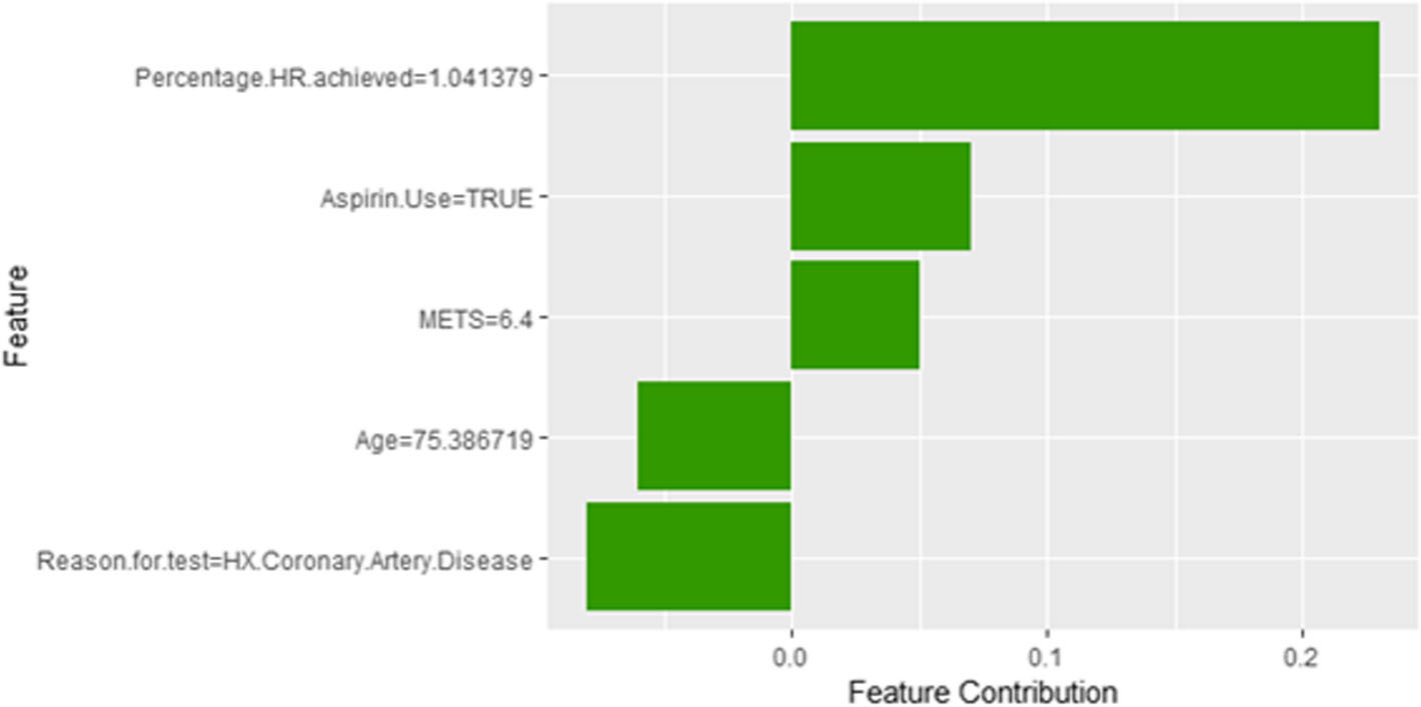
Shapley Values explanation of Instance 3 as False Positive Prediction of High Risk - Group 1 - Close to Maximum Age

Figure 12 shows LIME explanation of instance 3 based on *Age*, *Resting Systolic Blood Pressure*, *METS*, *Percentage HR achieved*, and *Peak Diastolic*. All the features used in the explanation positively contributed to the prediction of the high risk of hypertension with a probability equals to 0.68. Figure 13 shows the Shapley Values explanation of instance 3 based on *Percentage HR achieved*, *Aspirin Use*, *METS*, *Age*, and *Reason for test*. The most contributed feature toward increasing the probability high risk of hypertension is *Percentage HR achieved* while *Reason for test* is the most contributed feature toward decreasing the probability of the high risk of hypertension.

#### Instance 4 (False Positive Prediction of High Risk - Group 1 - Close to Minimum Age)

The description of this instance is as follows: *Age = 53.77, METS = 10.1, Resting Systolic Blood Pressure = 166, Peak Diastolic Blood Pressure = 90, Resting Diastolic Blood Pressure = 90, HX Coronary Artery Disease = false, Reason for test = Chest Pain, HX Diabetes = false, Percentage HR achieved = 0.93, Race = white, Hx Hyperlipidemia = true, Aspirin Use = false, Hypertension Response = true.*

**Fig. 14:**
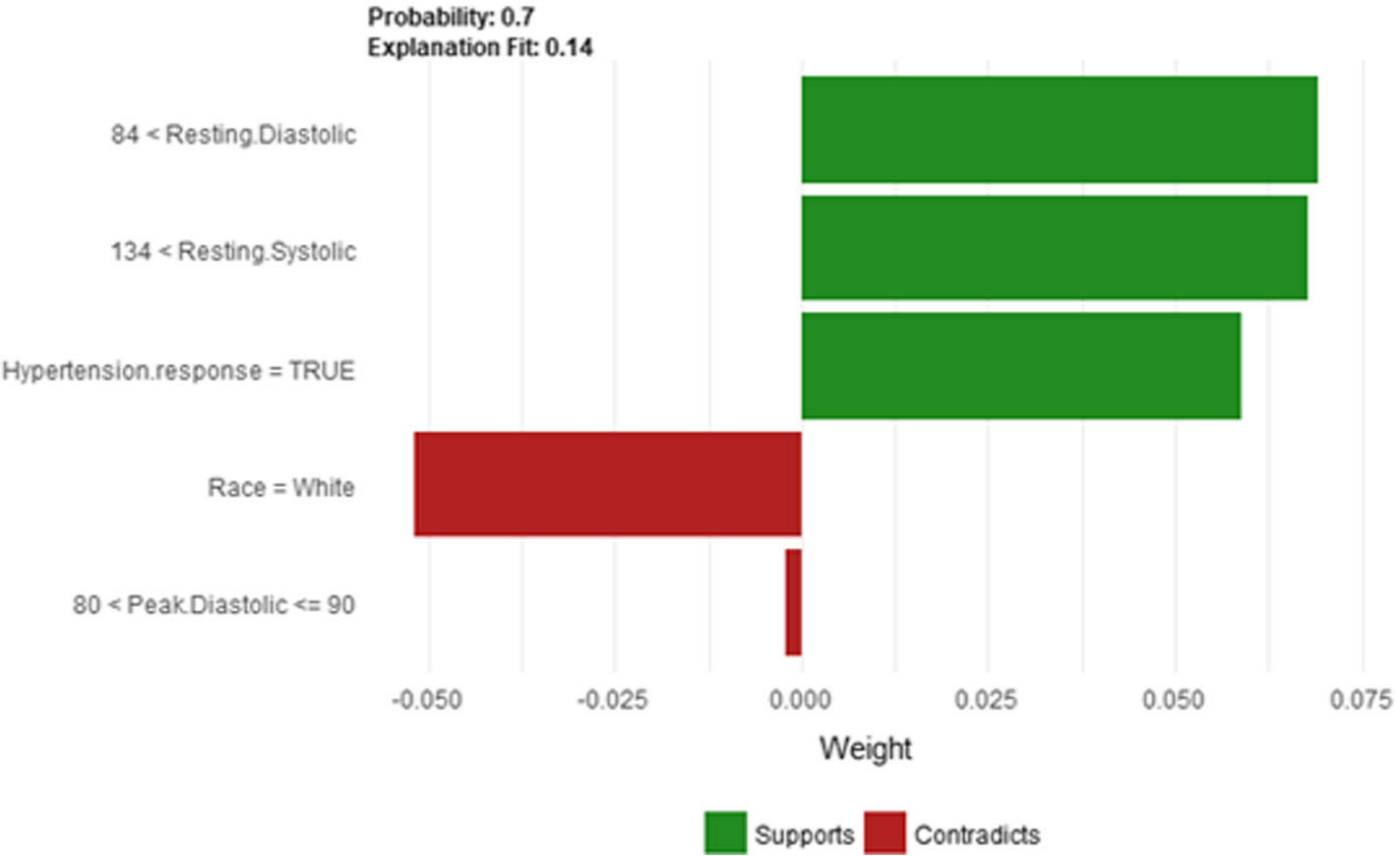
LIME explanation of Instance 4 as False Positive Prediction of High Risk - Group 1 - Close to Minimum Age

**Fig. 15:**
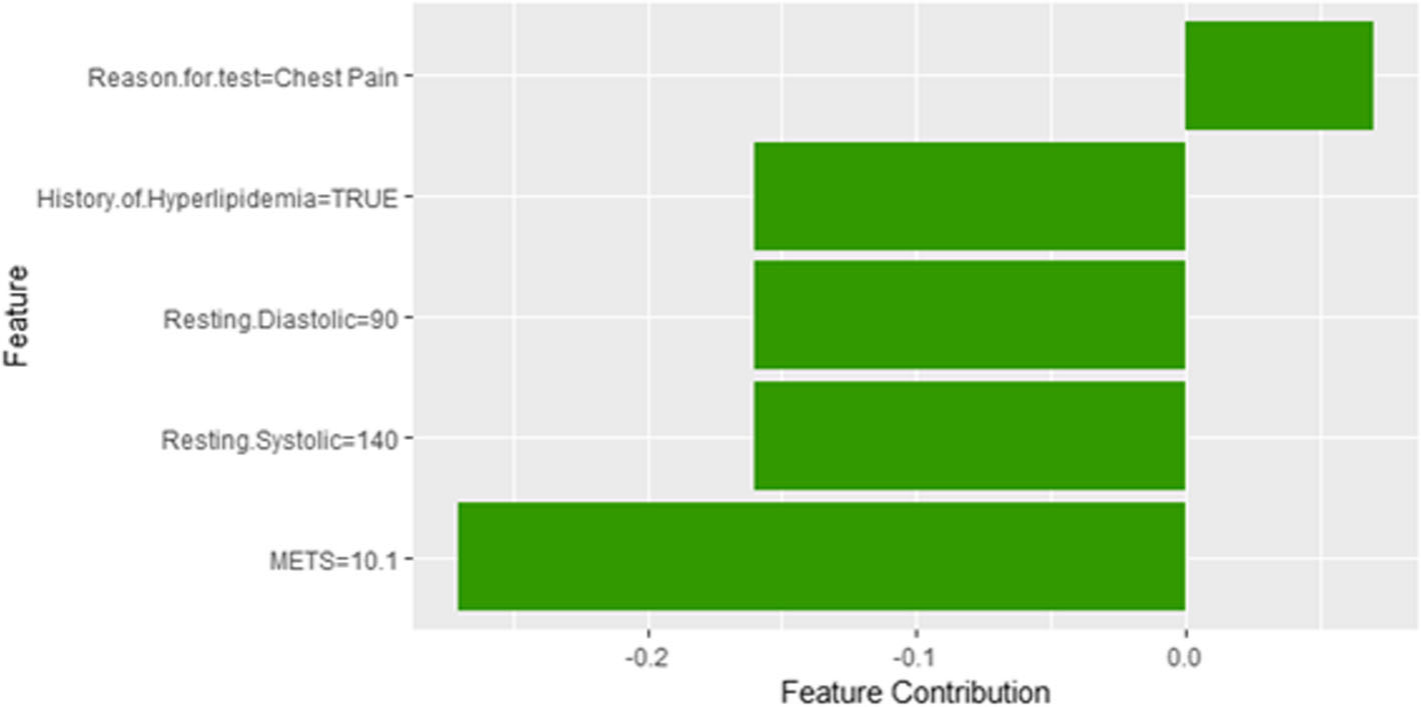
Shapley explanation of Instance 4 as False Positive Prediction of High Risk - Group 1 - Close to Minimum Age

Figure 14 shows LIME explanation of instance 4 as high risk of hypertension with a probability of 0.7. The explanation shows that *Resting Diastolic Blood Pressure*, *Resting Systolic Blood Pressure* and *Hypertension Response* are the most important features that positively strongly contributed to the prediction of high risk of hypertension while being white negatively contributed to the prediction of high risk of hypertension. Figure 15 shows Shapley Values explanation of instance 4 as high risk of hypertension based on *Reason for test*, *Hx Hyperlipidemia*, *Resting Diastolic Blood Pressure*, *Resting Systolic Blood Pressure* and *METS*. The most contributed feature toward increasing the probability high risk of hypertension is *Reason for test* while *METS* is the most contributed feature toward decreasing the probability of the high risk of hypertension.

#### Instance 5 (False Positive Prediction of High Risk - Group 1 - Close to Average Age)

The description of this instance is as follows: *Age = 67.9, METS = 6, Resting Systolic Blood Pressure = 114, Peak Diastolic Blood Pressure = 88, Resting Diastolic Blood Pressure = 78, HX Coronary Artery Disease = true, Reason for test = HX Coronary Artery Disease, HX Diabetes = false, Percentage HR achieved = 0.94, Race = white, Hx Hyperlipidemia = true, Aspirin Use = false, Hypertension Response = false*

**Fig. 16:**
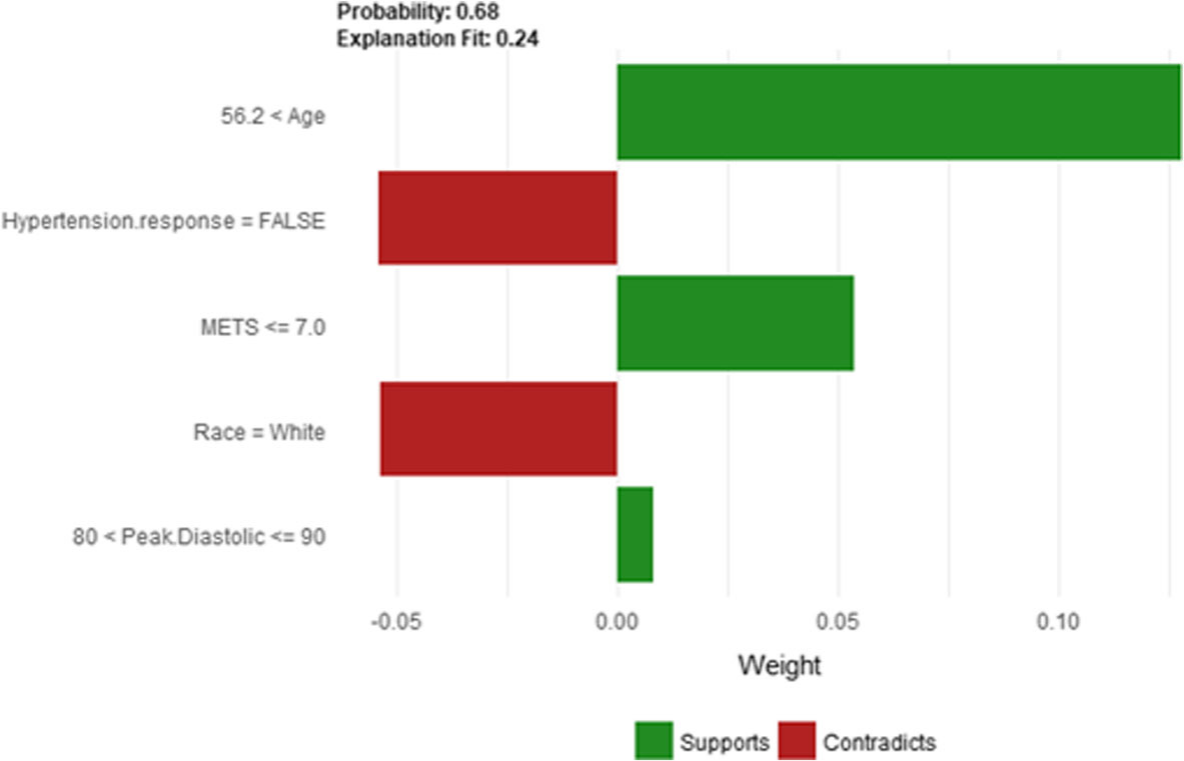
LIME explanation of Instance 5 as False Positive Prediction of High Risk - Group 1 - Close to Average Age

**Fig. 17:**
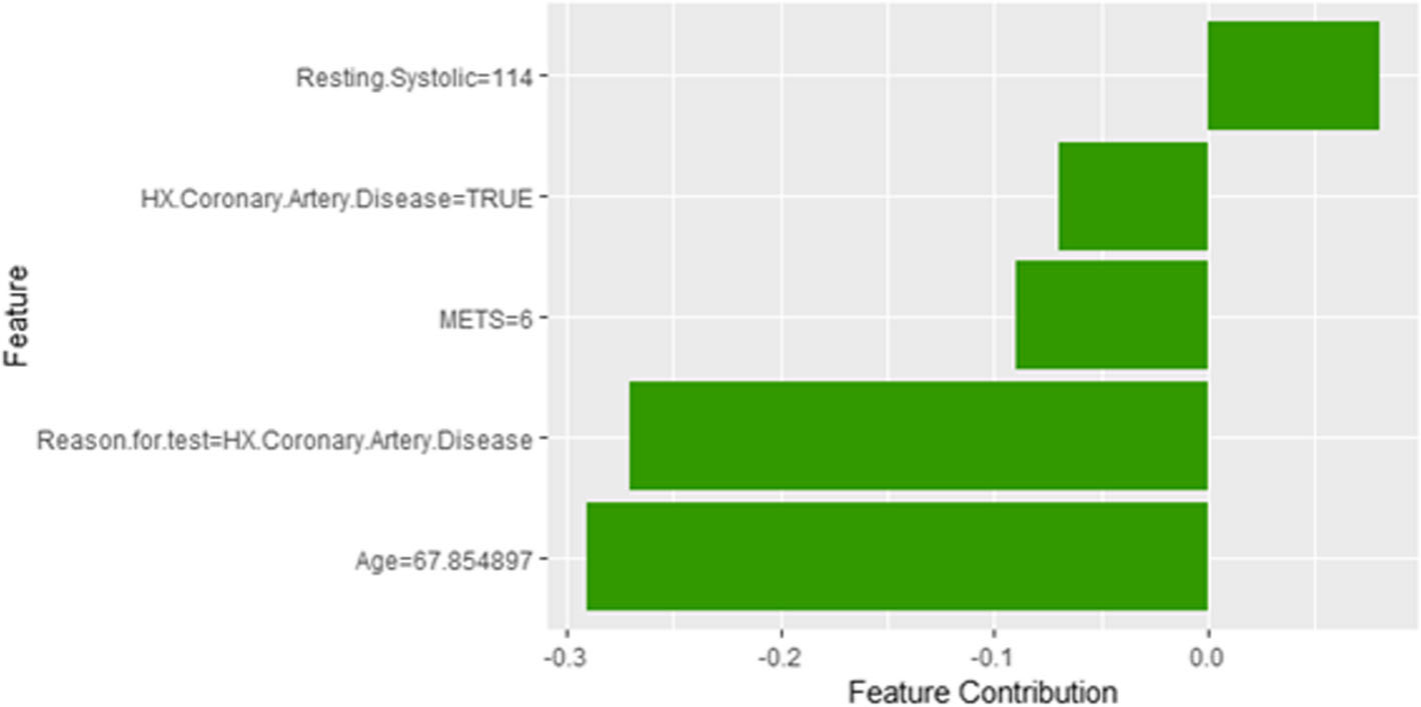
Shapley explanation of Instance 5 as False Positive Prediction of High Risk - Group 1 - Close to Average Age

The *Age* and *METS* are the most important features for LIME that positively contributed to the prediction of high risk of hypertension while being *white* and has negative *Hypertension Response test* negatively contributed to the prediction of high risk of hypertension as shown in Fig. 16. LIME explains instance 5 as high risk of hypertension with a probability of 0.68. Figure 17 shows Shapley Values explanation of instance 5 based on *Resting Systolic Blood Pressure*, *HX Coronary Artery Disease*, *METS*, *Reason for test* and *Age*. All the features except *Resting Systolic Blood Pressure* contributed toward decreasing the probability of the high risk of hypertension.

In the following, we present sample instances of *False Positive* predictions from *Group 2*. The instances are selected based on the patient’s age: one instance is close to the maximum age, one instance is close to the minimum age and one instance close to average age.

#### Instance 6 (False Positive Prediction of high Risk - Group 2 - Close to Maximum Age)

The description of this instance is as follows: *Age = 82.23, METS = 7, Resting Systolic Blood Pressure = 164, Peak Diastolic Blood Pressure = 80, Resting Diastolic Blood Pressure = 80, HX Coronary Artery Disease = false, Reason for test = Rule out Ischemia, HX Diabetes = false, Percentage HR achieved = 1.09, Race = white, Hx Hyperlipidemia = false, Aspirin Use = false, Hypertension Response = false*

**Fig. 18:**
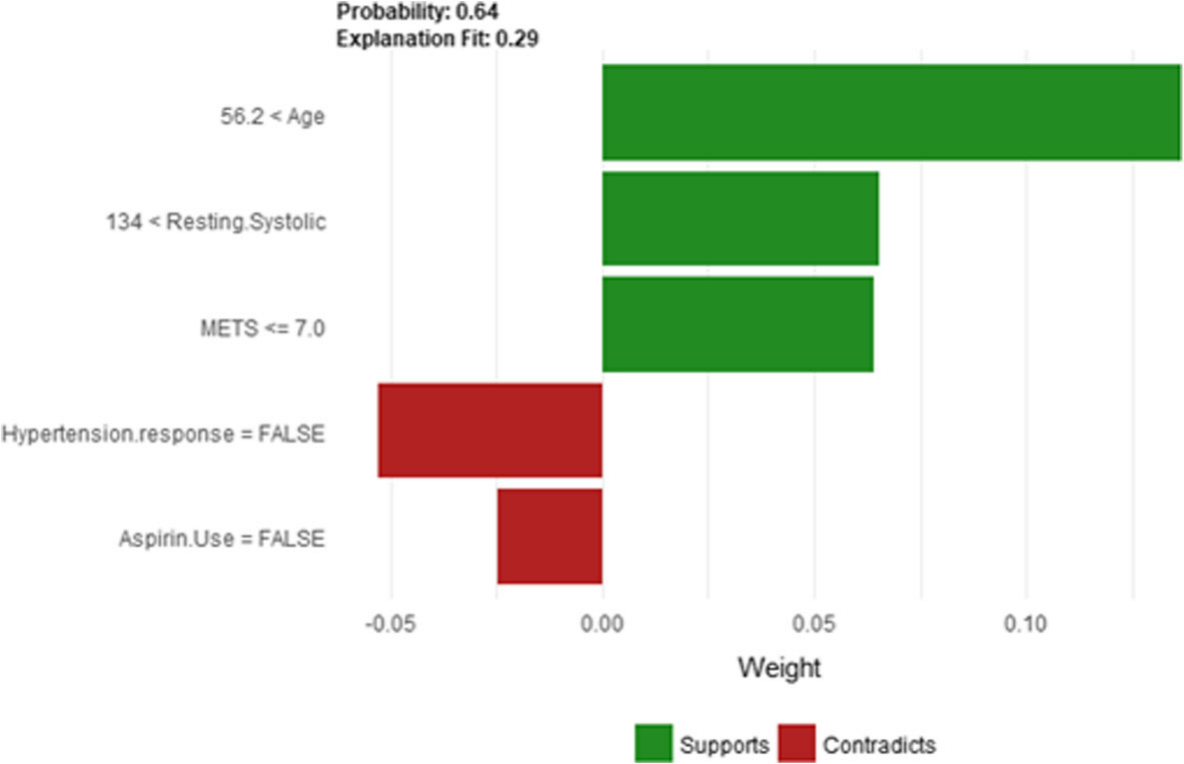
LIME explanation of instance 6 as False Positive Prediction of high Risk - Group 2 - Close to Maximum Age

**Fig. 19:**
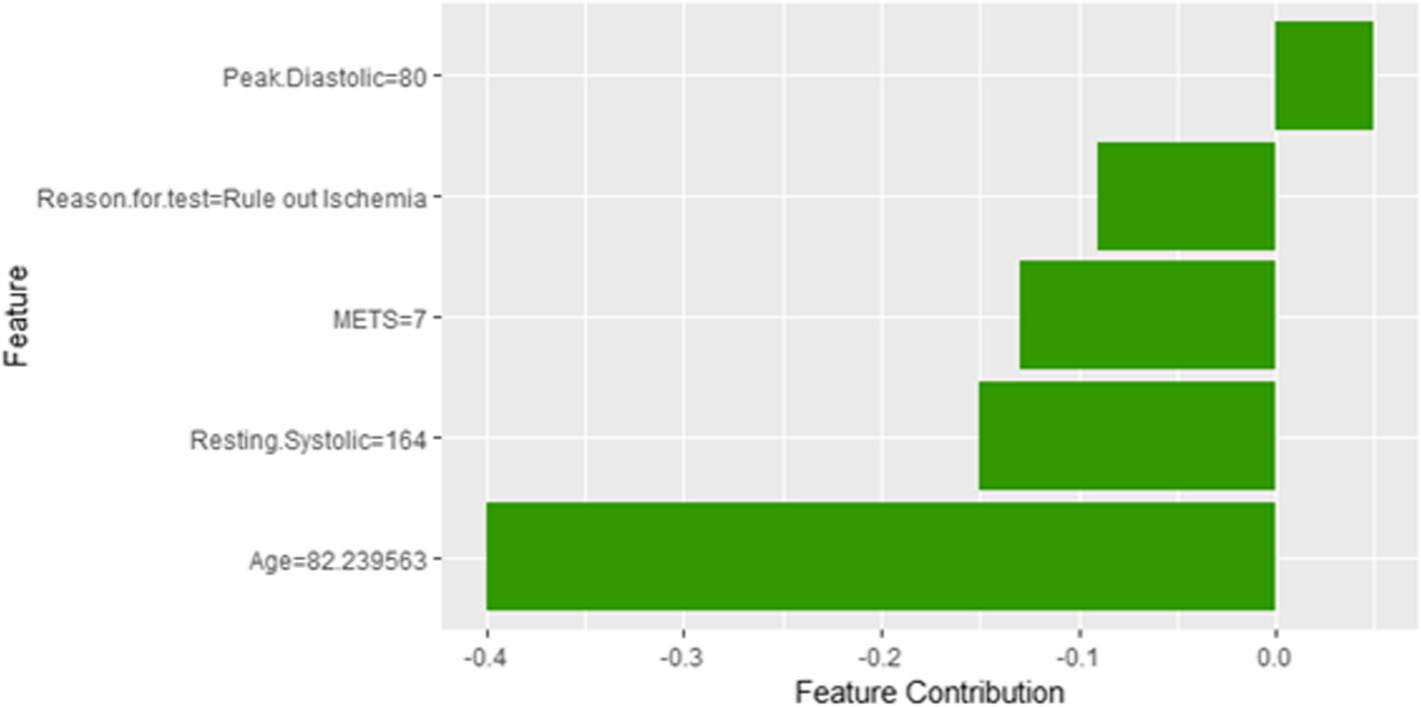
Shapley explanation of instance 6 as False Positive Prediction of high Risk - Group 2 - Close to Maximum Age

Figure 18 shows the explanation of instance 6 as high risk of hypertension with a weak probability of 0.64. The explanation is based on *Age*, *Resting Systolic Blood Pressure*, *METS*, *Hypertension Response,* and *Aspirin Use*. *Age*, *Resting Systolic Blood Pressure* and *METS* are positively contributed to the probability of high risk of hypertension while negative *Hypertension Response test* and not using *aspirin* are negatively contributed to the prediction of high risk of hypertension. Figure 19 shows the Shapley Values explanation of instance 6 as high risk of hypertension based on *Peak Diastolic Blood Pressure*, *Reason for test*, *METS*, *Resting Systolic Blood Pressure*, and *Age*. All the features except *Peak Diastolic Blood Pressure* contributed toward decreasing the probability of the high risk of hypertension

#### Instance 7 (False Positive Prediction of High Risk - Group 2 - Close to Minimum Age)

The description of this instance is as follows: *Age = 42.81, METS = 10, Resting Systolic Blood Pressure = 140, Peak Diastolic Blood Pressure = 98, Resting Diastolic Blood Pressure = 86, HX Coronary Artery Disease = false, Reason for test = shortness of breath, HX Diabetes = false, Percentage HR achieved = 0.92, Race = white, Hx Hyperlipidemia = true, Aspirin Use = false, Hypertension Response = true.*

**Fig. 20:**
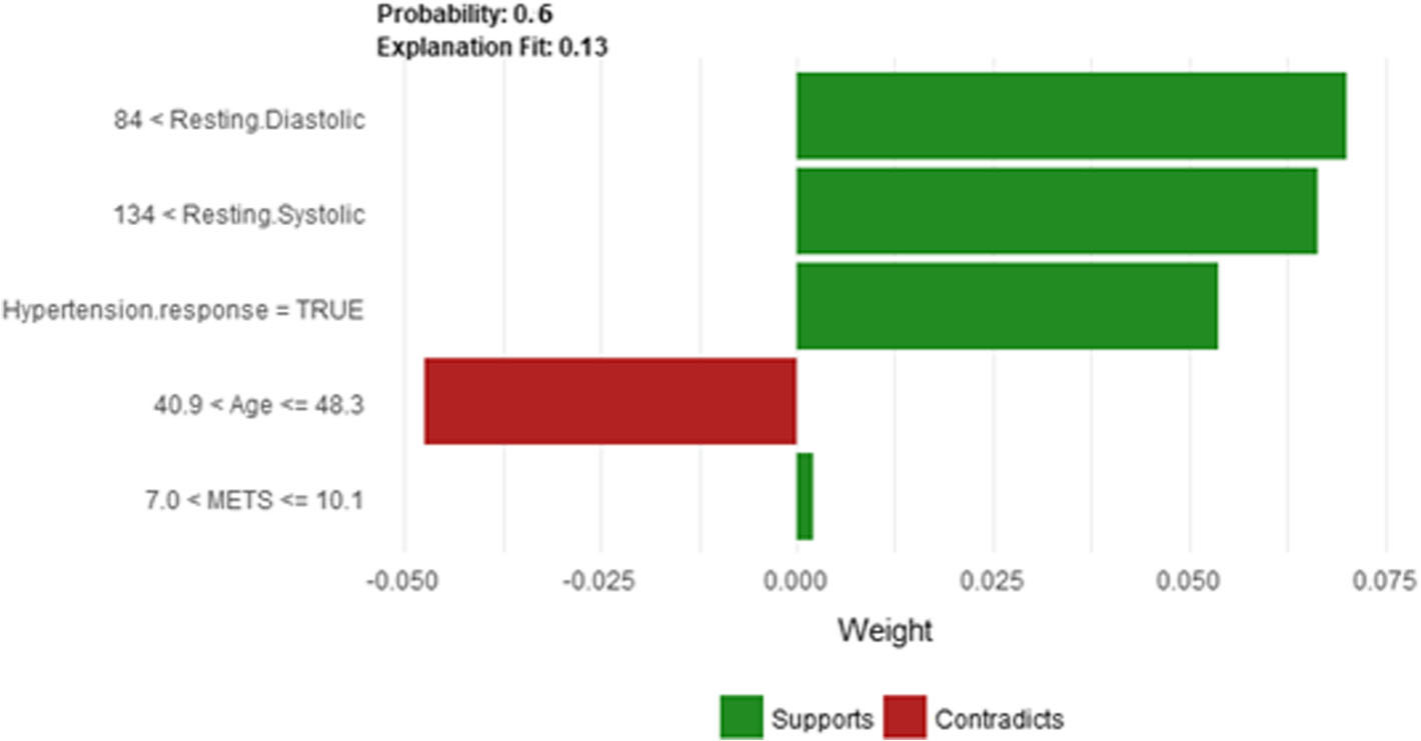
LIME explanation of Instance 7 as False Positive Prediction of High Risk - Group 2 - Close to Minimum Age

**Fig. 21:**
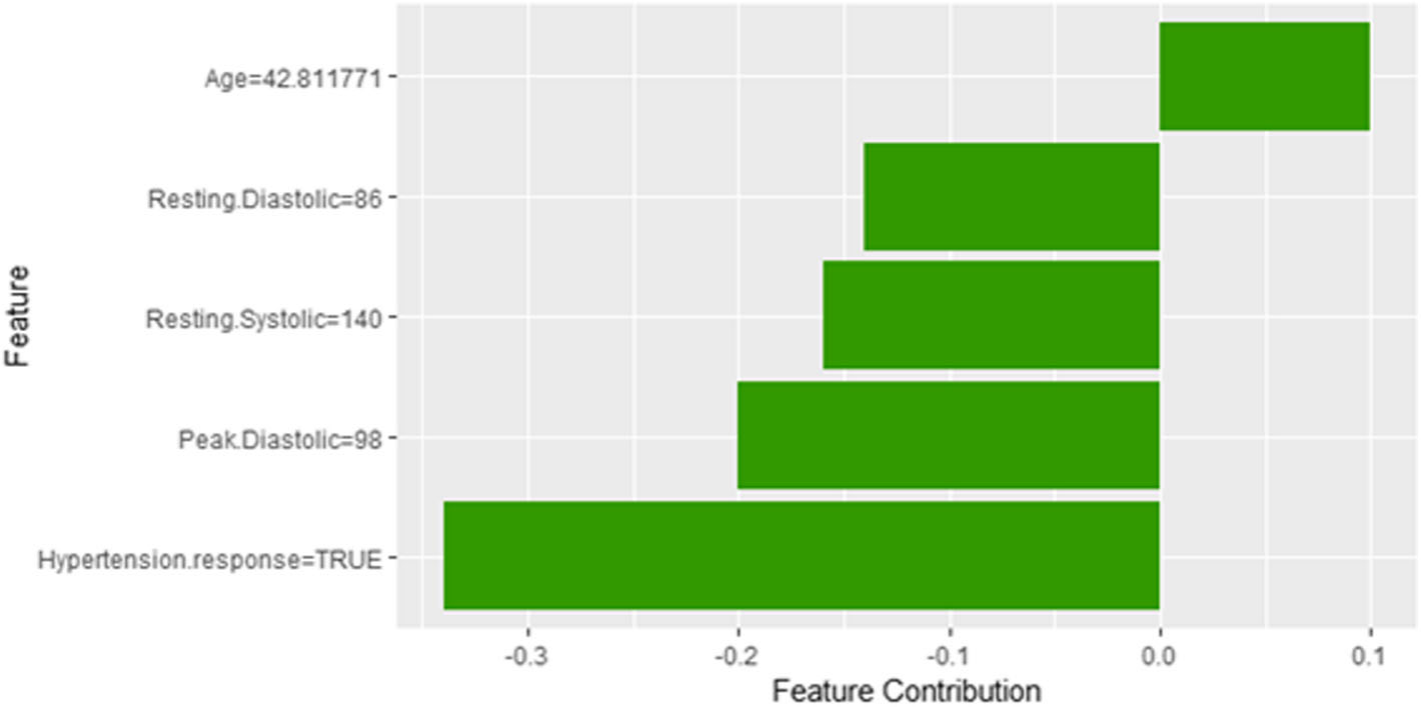
Shapely explanation of Instance 7 as False Positive Prediction of High Risk - Group 2 - Close to Minimum Age

Figure 20 shows LIME explanation of instance 7 as high risk of hypertension with a weak probability of 0.6. The explanation is based on *Resting Diastolic Blood Pressure*, *Resting Systolic Blood Pressure*, *Hypertension Response*, *Age* and *METS*. All the features used in the explanation except *Age* are positively contributed to the probability of high risk of hypertension. Figure 21 shows Shapley Values explanation of instance 7 as high risk of hypertension based on *Age*, *Resting Diastolic Blood Pressure*, *Resting Systolic Blood Pressure*, *Peak Diastolic Blood Pressure*, and *Hypertension Response*. All the features except *Age* contributed toward decreasing the probability of the high risk of hypertension.

#### Instance 8 (False Positive Prediction of High Risk - Group 2 - Close to Average Age)

The description of this instance is as follows: *Age* = 59.9, *METS* = 10.1, *Resting Systolic Blood Pressure* = 124, *Peak Diastolic Blood Pressure* = 90, *Resting Diastolic Blood Pressure* = 80, *HX Coronary Artery Disease* = false, *Reason for test* = chest pain, *HX Diabetes* = true, *Percentage HR achieved* = 0.675, *Race* = white, *Hx Hyperlipidemia* = false, *Aspirin Use* = false, *Hypertension Response* = false

**Fig. 22:**
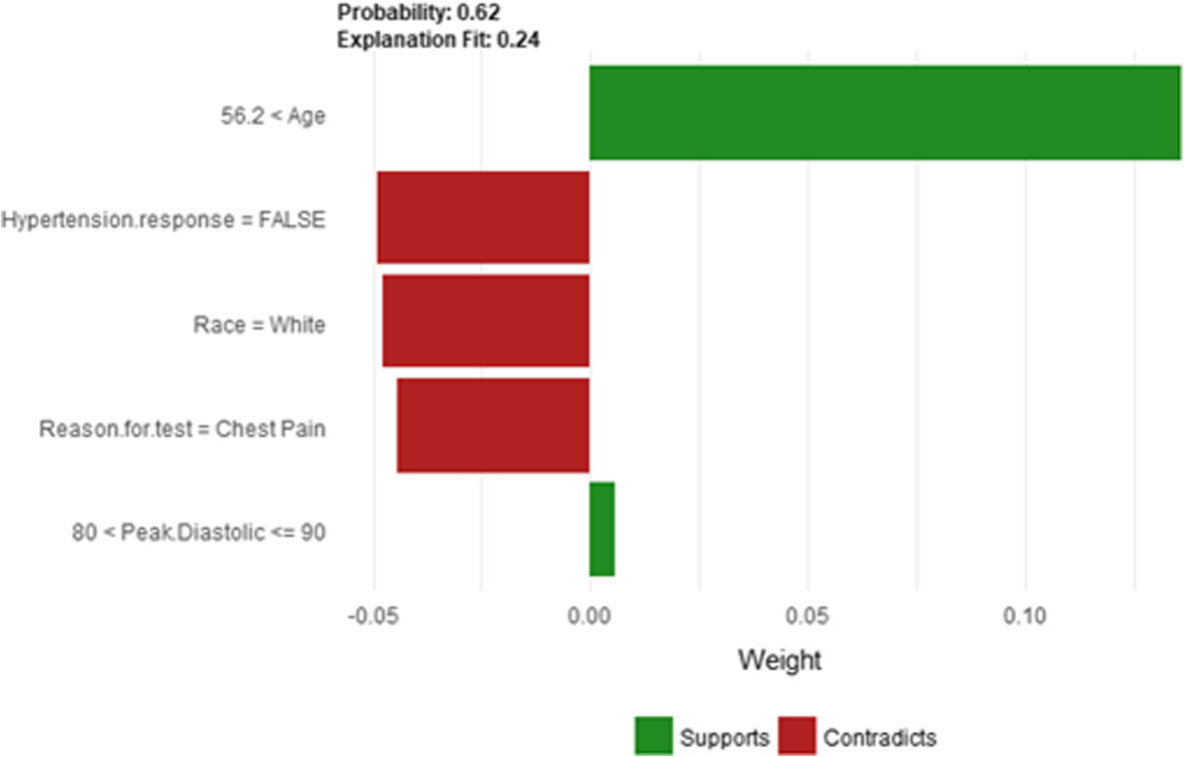
LIME explanation of Instance 8 as False Positive Prediction of High Risk - Group 2 - Close to Average Age

**Fig. 23:**
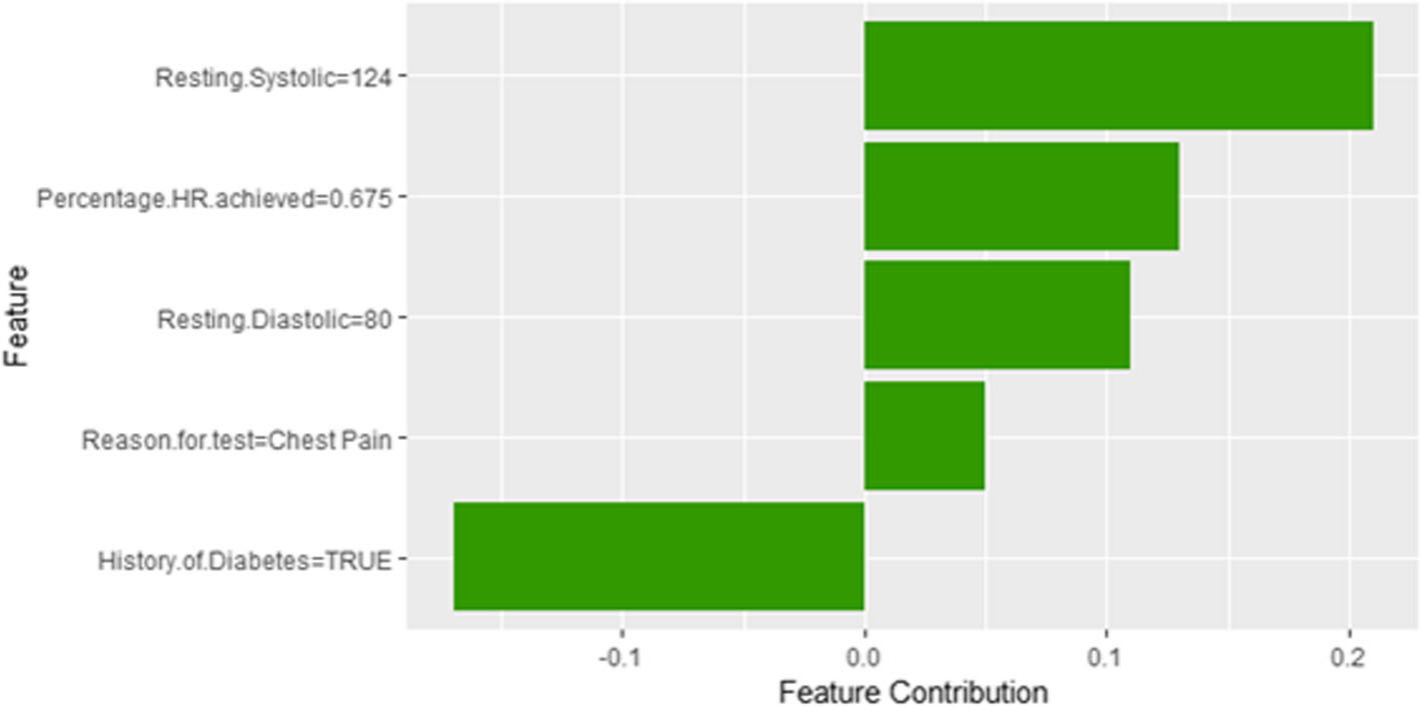
Shapley explanation of Instance 8 as False Positive Prediction of High Risk - Group 2 - Close to Average Age

Figure 22 shows LIME explanation of instance 8 based on *Age*, *Hypertension Response*, *Race*, *Reason for test* and *Peak Diastolic Blood Pressure*. *Age* and *Peak Diastolic Blood Pressure* contributed positively to the prediction of high risk of hypertension with a probability of 0:62, while *Hypertension Response*, *Race*, and *Reason for test* contributed negatively to the prediction of high risk of hypertension. Figure 23 shows Shapley Values explanation for instance 8 based on *Resting Systolic Blood Pressure*, *Percentage HR achieved*, *Resting Diastolic Blood Pressure*, *Reason for test*, and *HX Diabetes*. All the features except *HX Diabetes* contributed toward increasing the probability of the high risk of hypertension.

In the following, we present sample instances of *False Positive* predictions from *Group 3*. The instances are selected based on the patient’s age: one instance is close to the maximum age, one instance is close to the minimum age and one instance close to average age.

#### Instance 9 (False Positive Prediction of High Risk - Group 3 - Close to Maximum Age)

The description of this instance is as follows: *Age = 87.82, METS = 7, Resting Systolic Blood Pressure = 136, Peak Diastolic Blood Pressure = 80, Resting Diastolic Blood Pressure = 80, HX Coronary Artery Disease = 0, Reason for test = chest pain, HX Diabetes = 0, Percentage HR achieved = 1.098, Race = white, Hx Hyperlipidemia = true, Aspirin Use = false, Hypertension Response = false*.

**Fig. 24:**
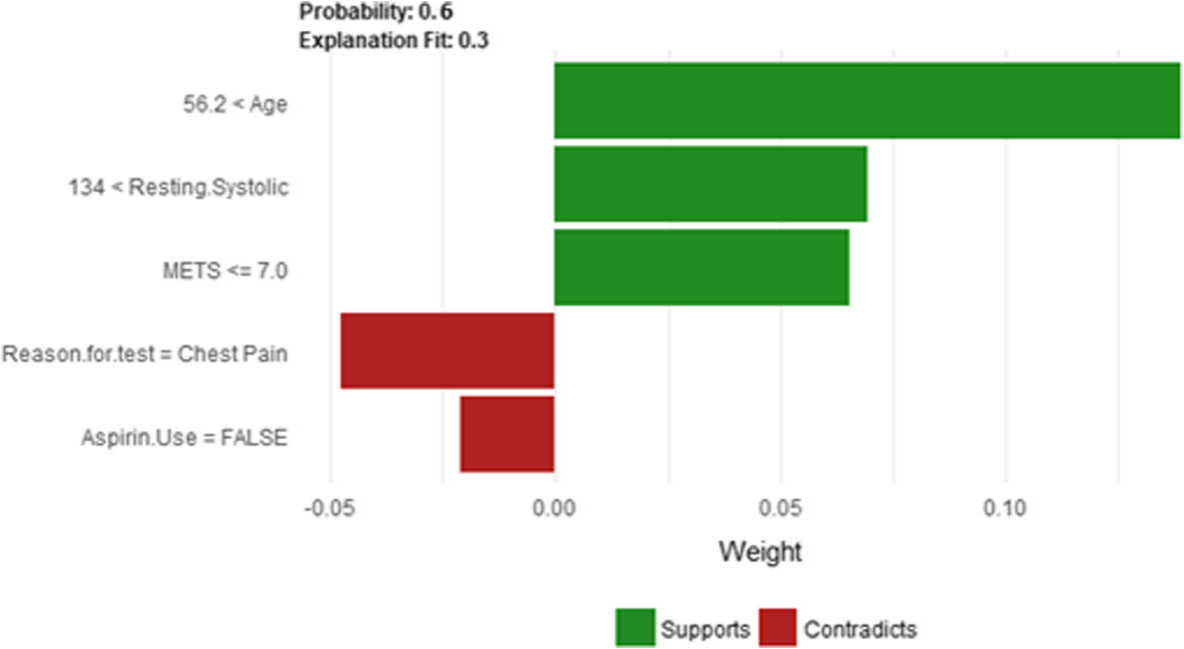
LIME explanation of Instance 9 as False Positive Prediction of High Risk - Group 3 - Close to Maximum Age

**Fig. 25:**
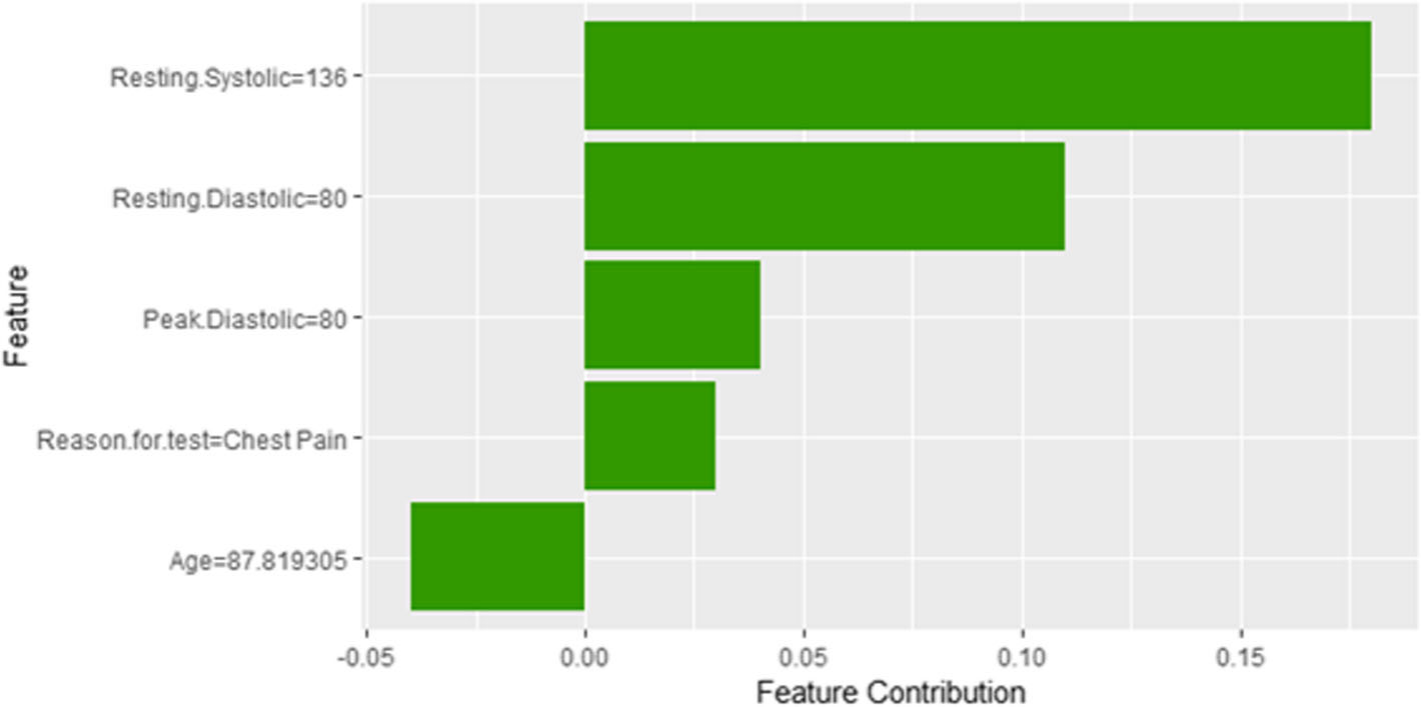
Shapley explanation of Instance 9 as False Positive Prediction of High Risk - Group 3 - Close to Maximum Age

Figure 24 shows LIME explanation of instance 9 based on *Age*, *Resting Systolic Blood Pressure*, METS, *Reason for test* and *Aspirin Use*. *Age*, *Resting Systolic Blood Pressure* and *METS* are the most contributed features for the prediction of the high risk of hypertension with a weak probability of 0.6. Figure 25 shows Shapley Values explanation of instance 9 based on *Resting Systolic Blood Pressure*, *Peak Diastolic Blood Pressure*, *Reason for test* and *Age*. All the features except *Age* contributed toward increasing the probability of the high risk of hypertension.

#### Instance 10 (False Positive Prediction of High Risk - Group 3 - close to Minimum Age)

The description of this instance is as follows: *Age = 29.13, METS = 5, Resting Systolic Blood Pressure = 148, Peak Diastolic Blood Pressure = 60, Resting Diastolic Blood Pressure = 92, HX Coronary Artery Disease = 0, Reason for test = Chest Pain, HX Diabetes = 0, Percentage HR achieved = 0.79, Race = black, Hx Hyperlipidemia = false, Aspirin Use = false, Hypertension Response = false*.

**Fig. 26:**
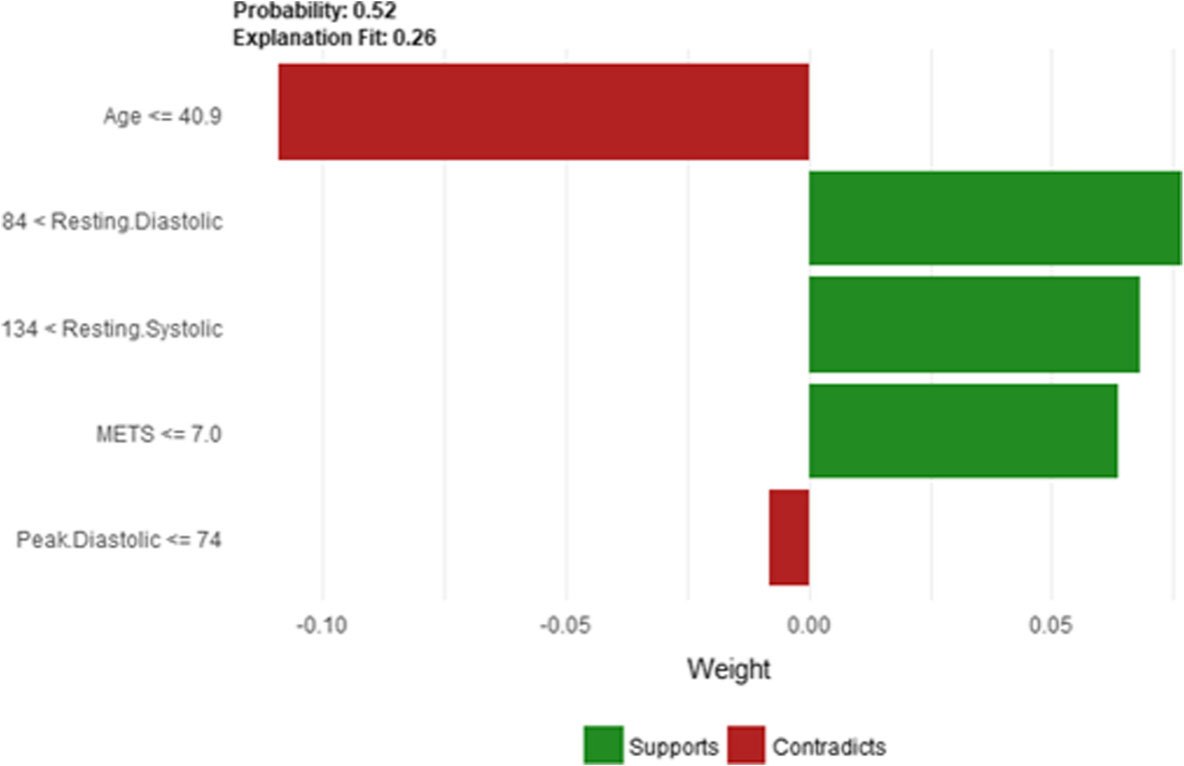
LIME explanation of Instance 10 as False Positive Prediction of High Risk - Group 3 - close to Minimum Age

**Fig. 27:**
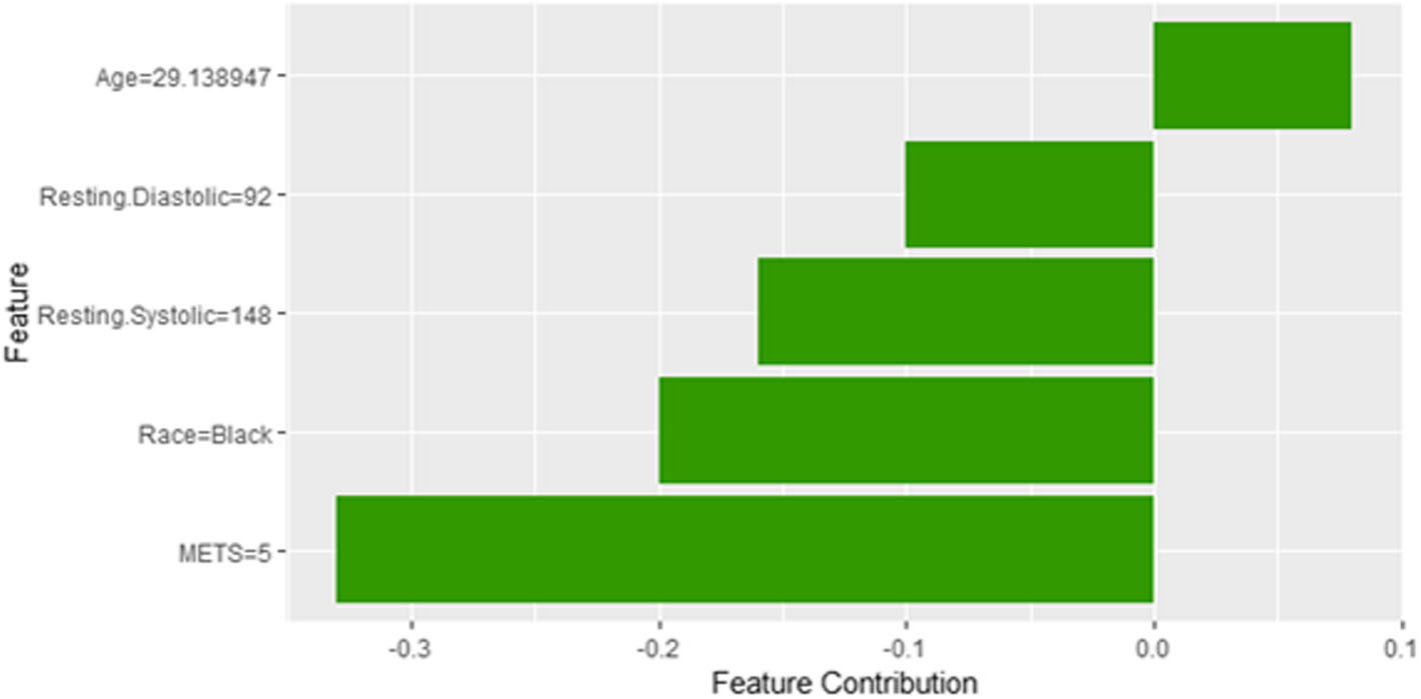
Shapley explanation of Instance 10 as False Positive Prediction of High Risk - Group 3 - close to Minimum Age

Instance 10 is incorrectly predicted by the black box model as a high risk of hypertension with a weak probability equals to 0.52 using LIME explainer as shown in Fig. 26. It is clear from the explanation that the young *Age* of the patient strongly contributed against the prediction of the high risk of hypertension while *Resting Diastolic Blood Pressure*, *Resting Systolic Blood Pressure* and *METS* contributed positively to the prediction of the high risk of hypertension. The explanation of instance 10 using Shapley Values is shown in Fig. 27 using features *Age*, *Resting Diastolic Blood Pressure*, *Resting Systolic Blood Pressure*, *Race* and *METS*. The feature *Age* is the only features contributed toward increasing the probability of high risk of hypertension.

#### Instance 11 (False Positive Prediction of High Risk - Group 3 - Close to Average Age)

The description of this instance is as follows: *Age = 56.4, METS = 7, Resting Systolic Blood Pressure = 138, Peak Diastolic Blood Pressure = 60, Resting Diastolic Blood Pressure = 82, HX Coronary Artery Disease = false, Reason for test = Screening, HX Diabetes = false, Percentage HR achieved = 0.87, Race = white, Hx Hyperlipidemia = false, Aspirin Use = false, Hypertension Response = false*.

**Fig. 28:**
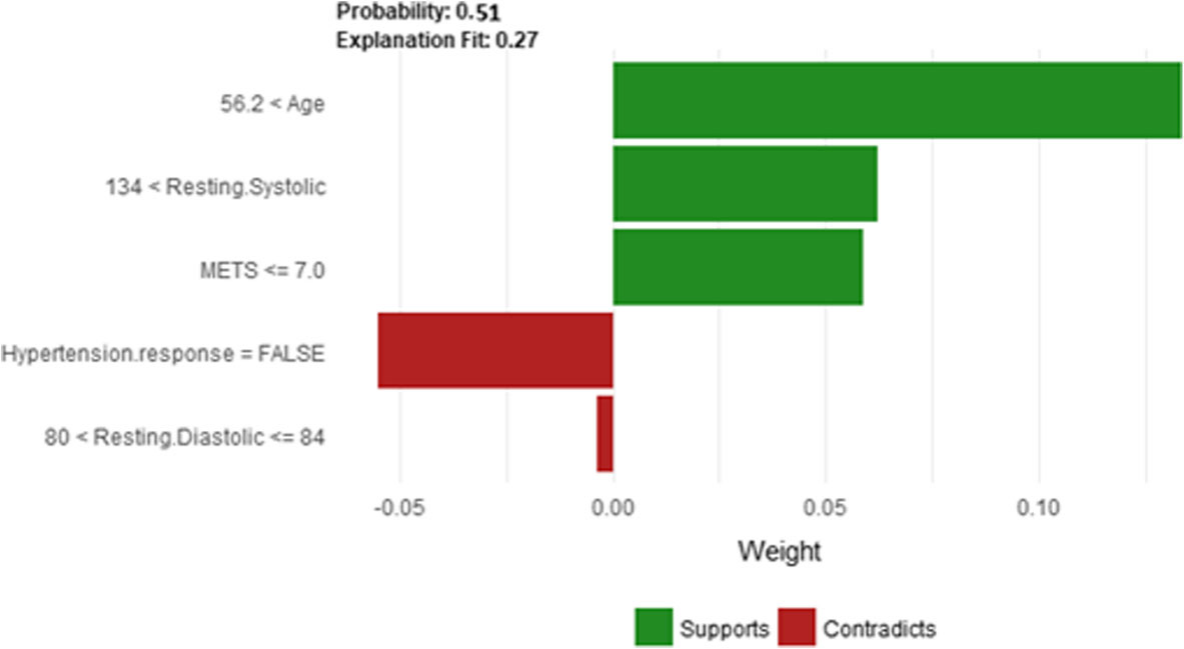
LIME explanation of Instance 11 as False Positive Prediction of High Risk - Group 3 - Close to Average Age

**Fig. 29:**
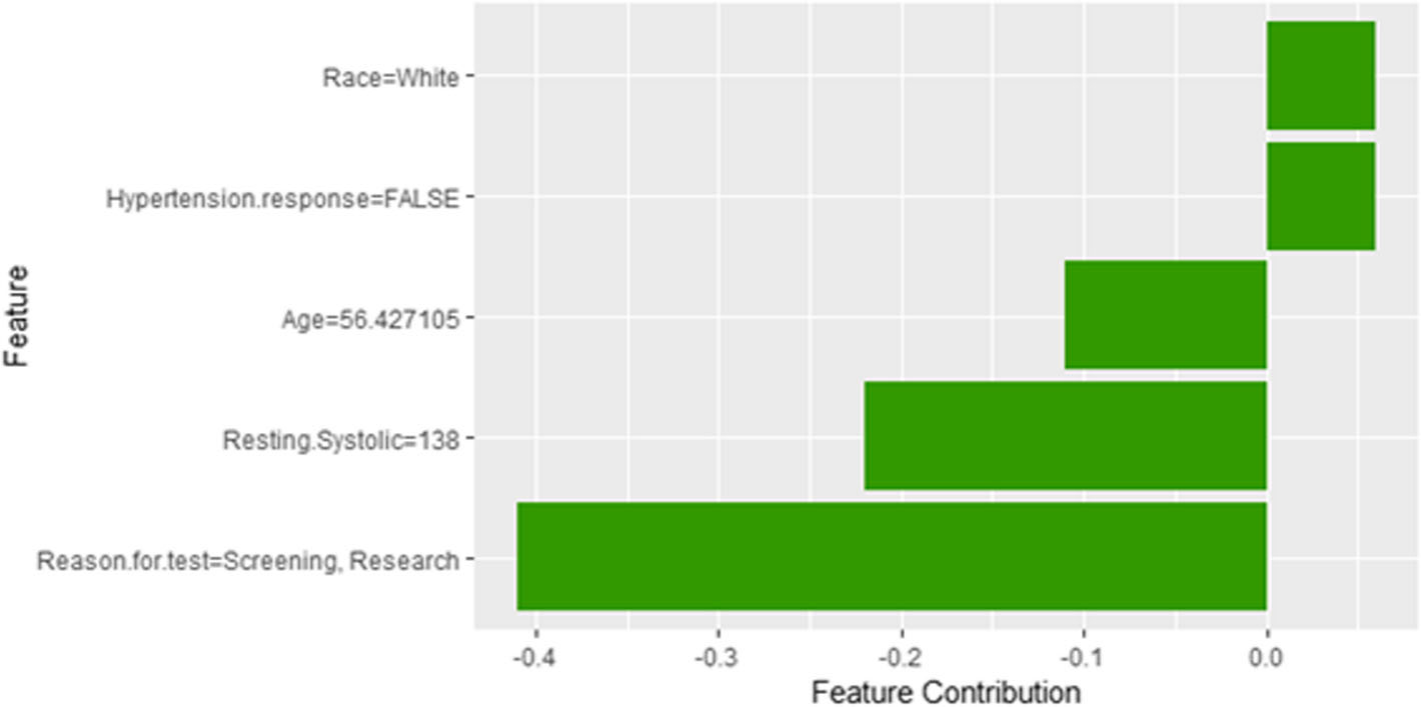
Shapley explanation of Instance 11 as False Positive Prediction of High Risk - Group 3 - Close to Average Age

Figure 28 shows LIME explanation of instance 11 as a high risk of hypertension with a probability of 0.51. Features *Age*, *Resting Systolic Blood Pressure* and *METS* are the main features that contributed to the prediction of the high risk of hypertension. Shapley Values explanation for instance 11 is shown in Fig. 29, based on *Race*, *Hypertension Response*, *Age*, *Resting Systolic Blood Pressure*, and *Reason for test*. The two features *Race* and *Hypertension Response* are the only features contributed toward the increasing probability of high risk of hypertension. The explanations of these False Positive examples show that the *Age* is the most influencing feature towards the explanation of the high risk of hypertension based on LIME. We noticed that instances in *Group 3* have the lowest average age of 56, while instances in *Group 1* has the highest average age of 68 amongst the three groups which clearly indicates that the probability of low risk of hypertension decreases with the increase in the patient’s age.

**Fig. 30:**
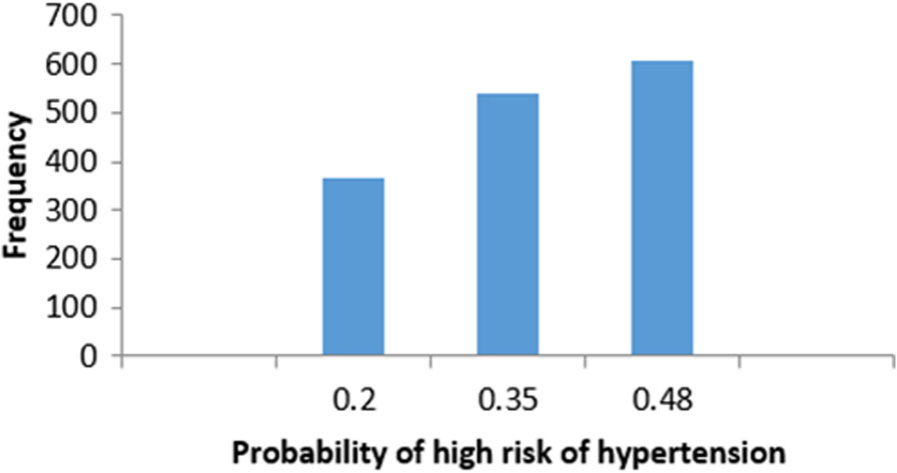
Histogram of false negative instances

In the following, we are going to have a deep look at examples for instances that have *False Negative* predications (Incorrectly classified as low risk of hypertension). Figure 30 shows the frequency distribution of the false negative instances based on the probability of high risk of hypertension. The probability of high risk of hypertension has been split into another three groups. **Group 4** represents instances with the probability of high risk of hypertension between [0–0.2]. **Group 5** and Group 6 represent instances with a probability of high risk of hypertension belongs to]0.2–0.35] and]0.35–0.48[, respectively (0.48 is the highest probability in the *False Negative* instances). In particular, we present sample instances of *False Negative* predictions from *Group 4*. The instances are selected based on the patient’s age: one instance is close to the maximum age, one instance is close to the minimum age and one instance close to average age.

#### Instance 12 (False Negative Prediction of Low Risk - Group 4 - Close to Maximum Age)

The description of this instance is as follows: *Age = 63.8, METS = 13, Resting Systolic Blood Pressure = 112, Peak Diastolic Blood Pressure = 80, Resting Diastolic Blood Pressure = 72, HX Coronary Artery Disease = false, Reason for test = Rule out Ischemia, HX Diabetes = false, Percentage HR achieved = 0.95, Race = white, Hx Hyperlipidemia = false, Aspirin Use = false, Hypertension Response = false.*

**Fig. 31:**
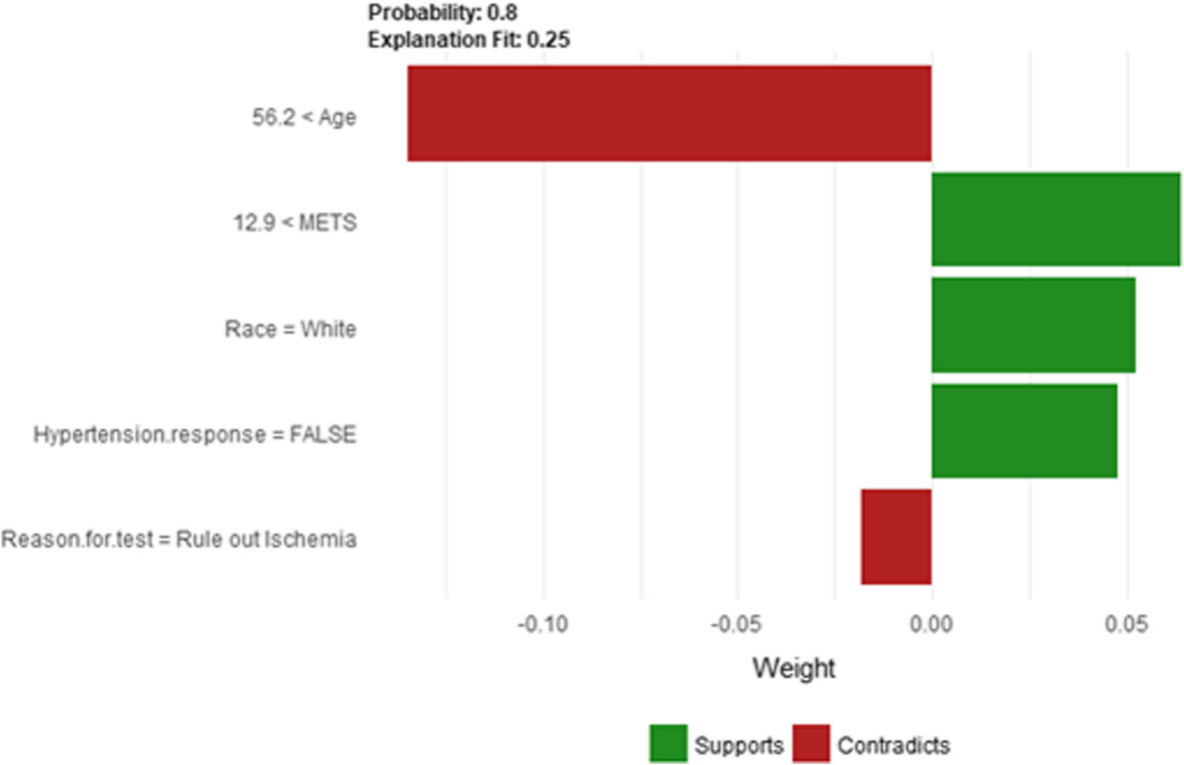
LIME explanation of Instance 12 as False Negative Prediction of Low risk - Group 4 - Close to Maximum Age

**Fig. 32:**
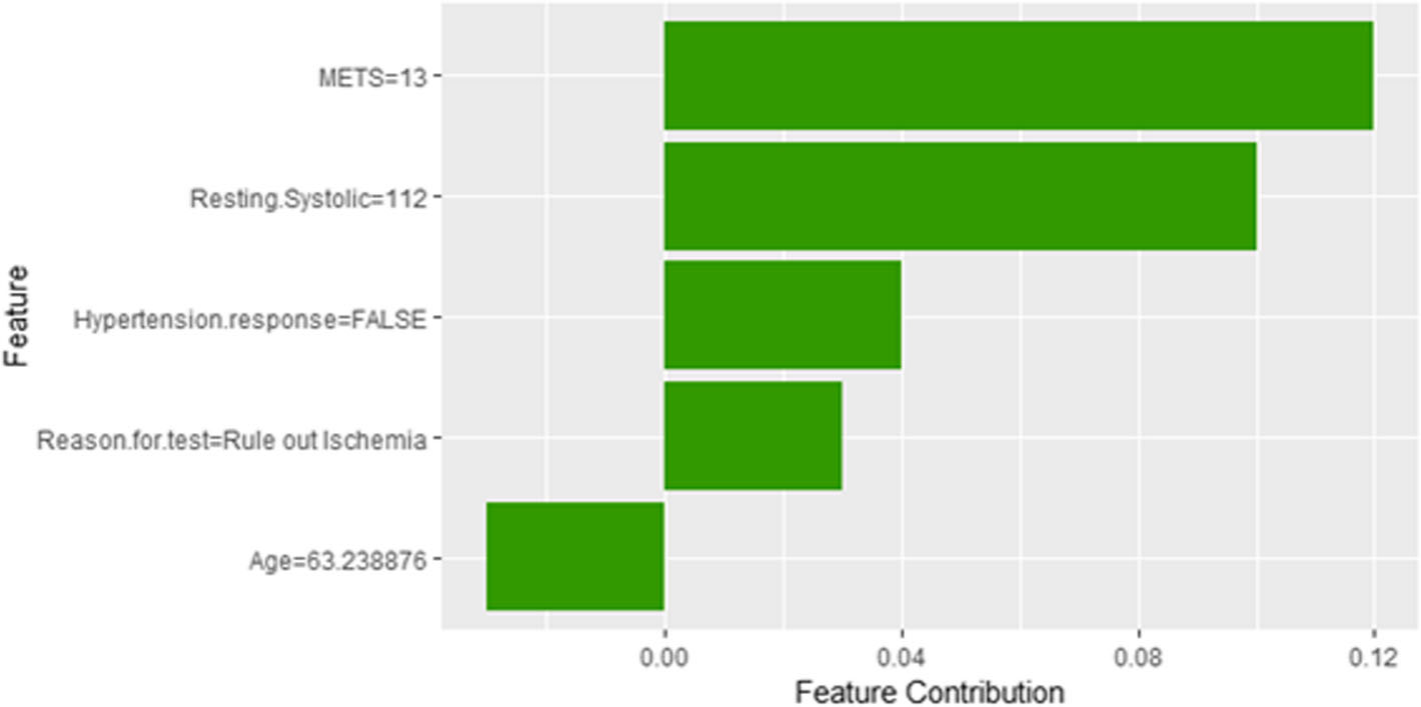
Shapley explanation of Instance 12 as False Negative Prediction of Low risk - Group 4 - Close to Maximum Age

Figure 31 shows the explanation of instance 12 as low risk of hypertension with a strong probability of 0.8. The explanation is based on *Age*, *METS*, *Race*, *Hypertension Response* and *Reason for test*. *Age* is the most influencing feature that negatively contributed to the prediction of low risk of hypertension while *METS*, *Race* and *Hypertension Response* contributed positively to the prediction of low risk of hypertension. Figure 32 shows Shapley values explanation for instance 12 based on *METS*, *Resting Systolic Blood Pressure*, *Hypertension Response*, *Reason* for *test*, and *Age*. Similar to LIME explanation, features *METS,* and *Hypertension Response* contributed toward the probability of low risk of hypertension.

#### Instance 13 (False Negative Prediction of Low Risk - Group 4 - Close to Minimum Age)

The description of this instance is as follows: *Age = 18.8, METS = 15, Resting Systolic Blood Pressure = 120, Peak Diastolic Blood Pressure = 90, Resting Diastolic Blood Pressure = 80, HX Coronary Artery Disease = false, Reason for test = Chest Pain,HX Diabetes = 0, Percentage HR achieved = 0.85, Race = black, Hx Hyperlipidemia = false, Aspirin Use = false, Hypertension Response = false*.

**Fig. 33:**
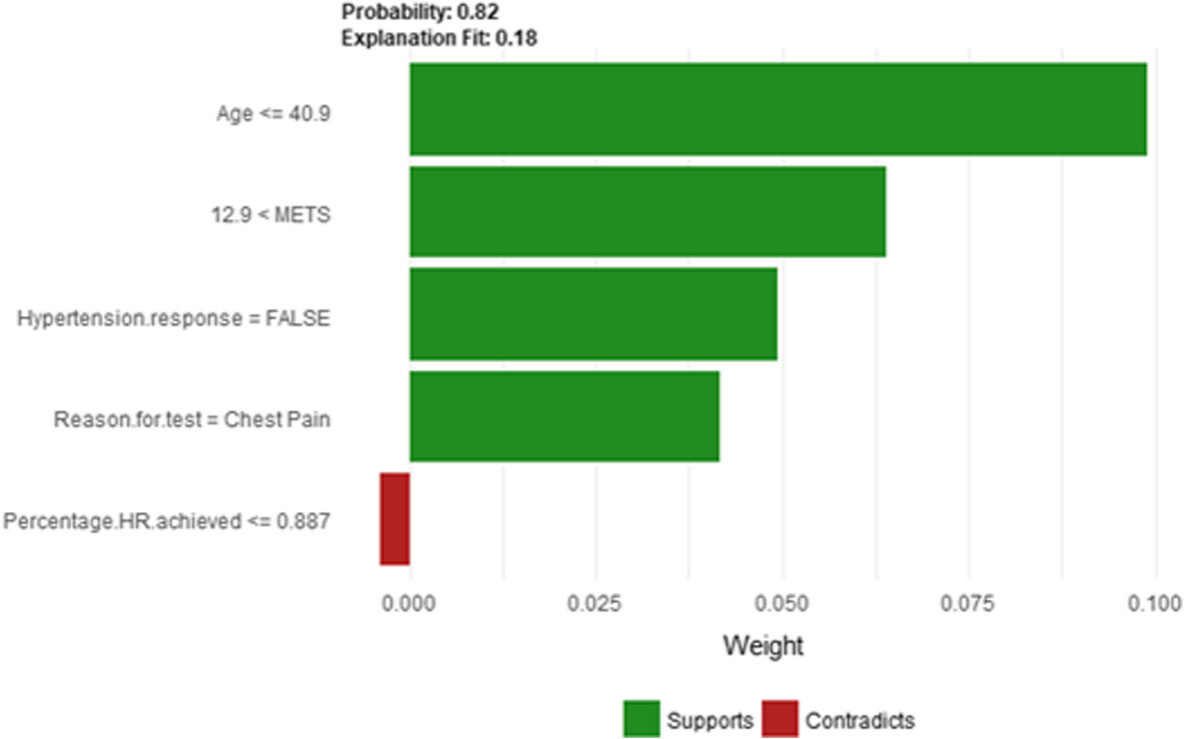
LIME explanation of Instance 13 as False Negative Prediction of Low Risk - Group 4 - Close to Minimum Age

**Fig. 34:**
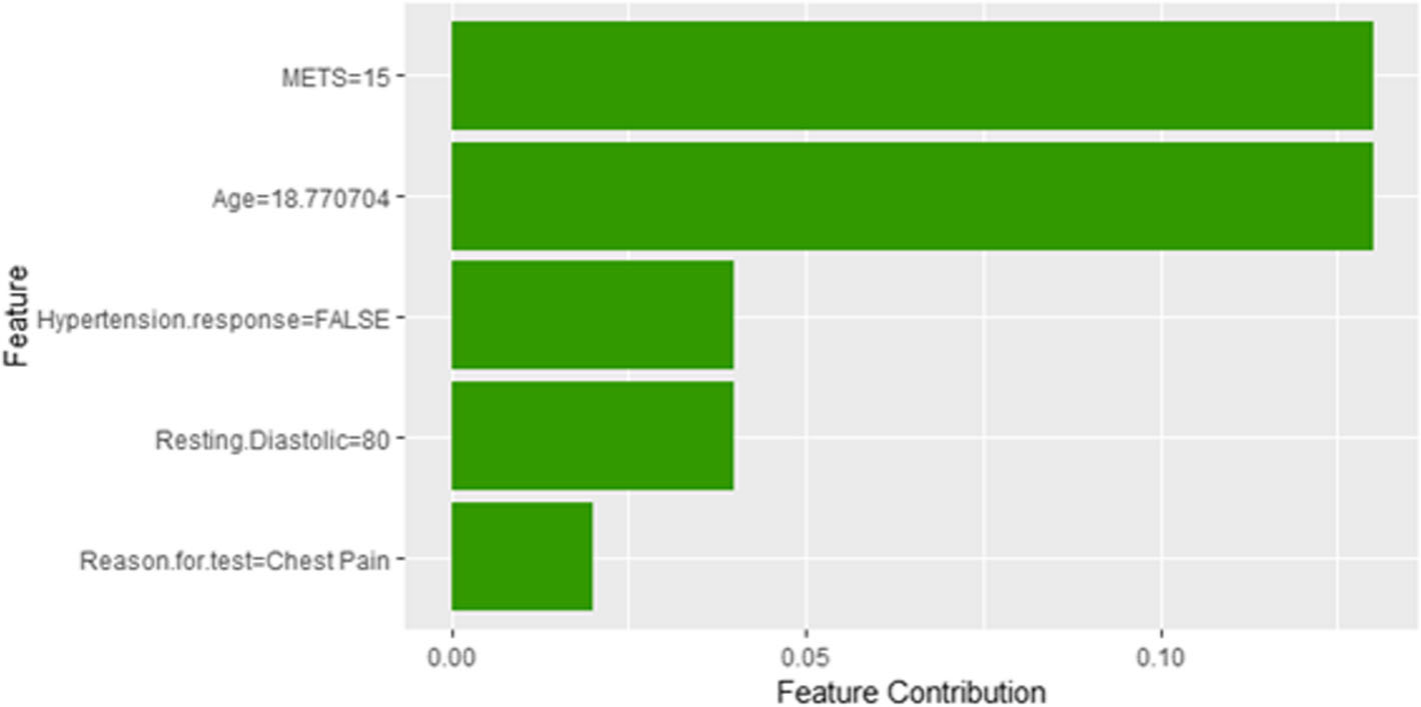
Shapley explanation of Instance 13 as False Negative Prediction of Low Risk - Group 4 - Close to Minimum Age

Figure 33 shows the explanation of instance 13 based on *Age*, *METS*, *Hypertension Response*, *Reason for test* and *Percentage HR achieved*. All the features used in the explanation except *Percentage HR achieved* contributed positively to the prediction of low risk of hypertension (probability = 0.82). Figure 34 shows Shapley Values explanation for instance 13 based on *Age*, *Reason* for *test*, *Resting Diastolic Blood Pressure*, *Hypertension Response*, *METS*. All the features in the explanation contributed toward the probability of low risk of hypertension

#### Instance 14 (False Negative Prediction of Low risk - Group 4 - Close to Average Age)

The description of this instance is as follows: *Age = 48.26, METS = 12, Resting Systolic Blood Pressure = 110, Peak Diastolic Blood Pressure = 70, Resting Diastolic Blood Pressure = 70, HX Coronary Artery Disease = false, Reason for test = Chest Pain, HX Diabetes = false, Percentage HR achieved = 0.85, Race = white, Hx Hyperlipidemia = false, Aspirin Use = false, Hypertension Response = false*.

**Fig. 35:**
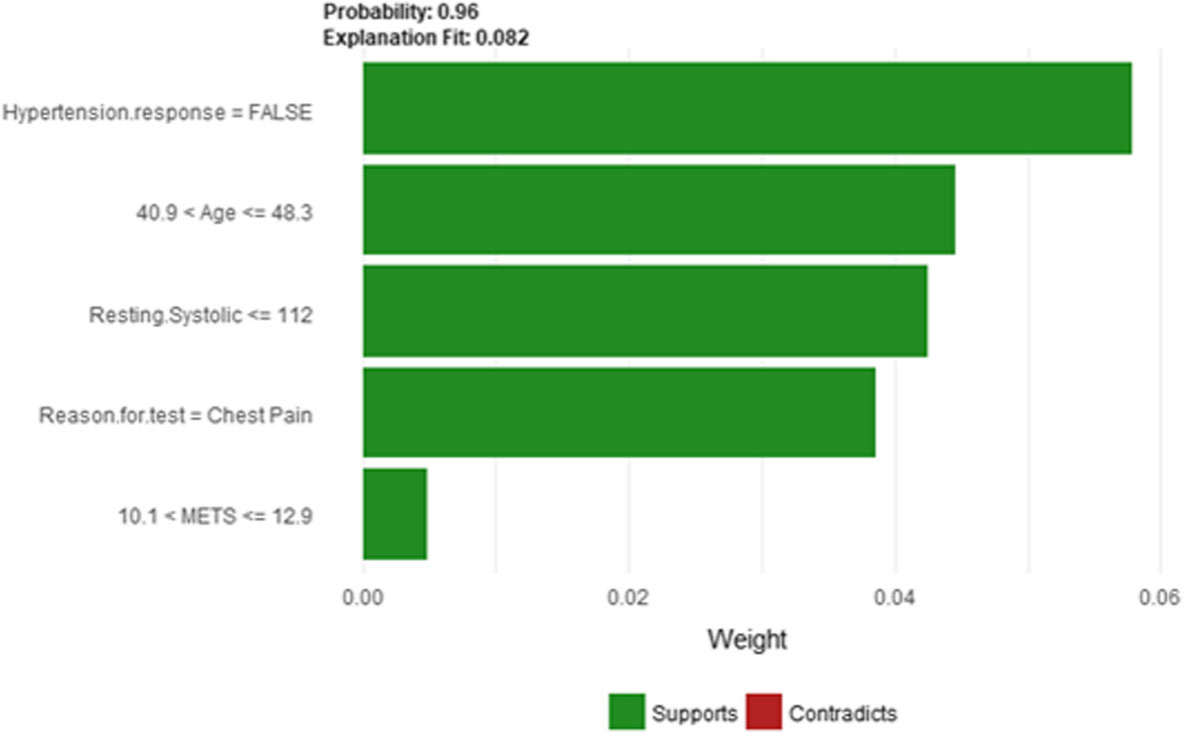
LIME explanation of Instance 14 as False Negative Prediction of Low risk - Group 4 - Close to Average Age

**Fig. 36:**
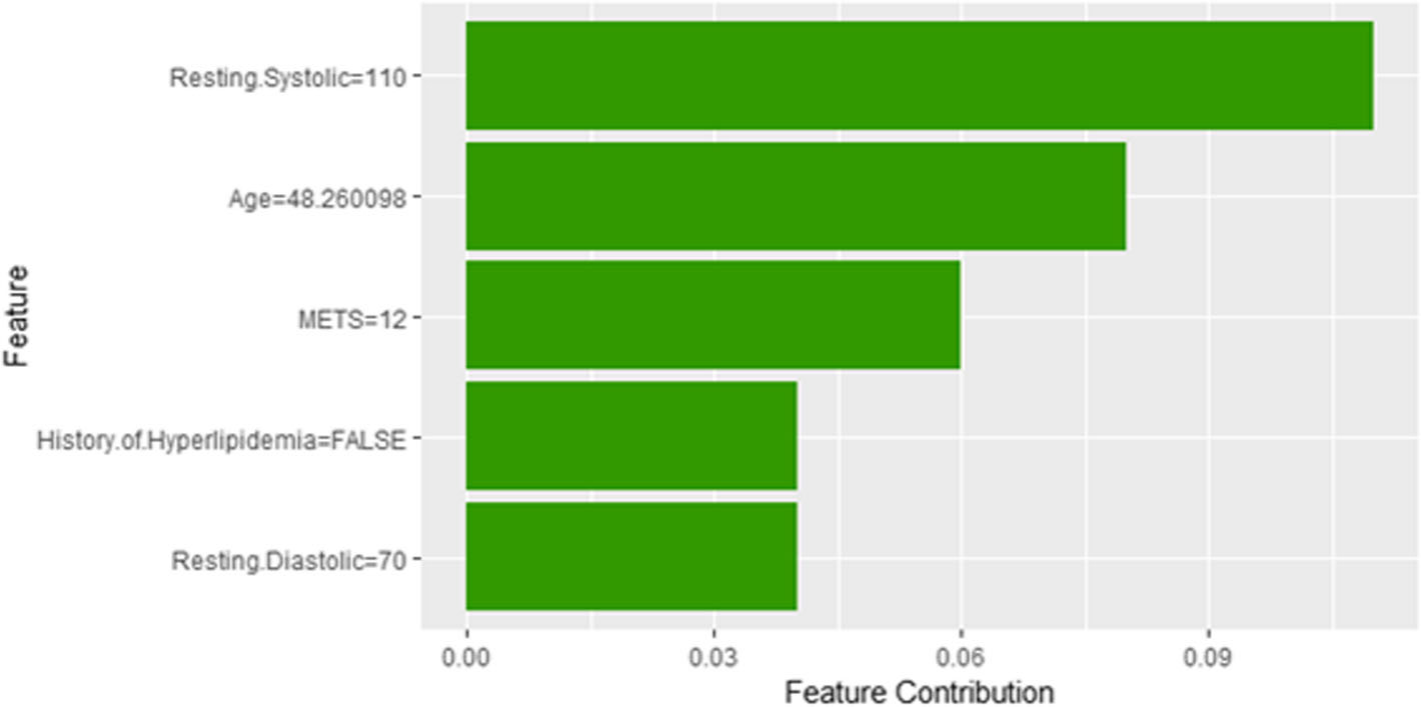
Shapley explanation of Instance 14 as False Negative Prediction of Low risk - Group 4 - Close to Average Age

Figure 35 shows LIME explanation of instance 14 based on *Hypertension Response*, *Age*, *Resting Systolic Blood Pressure*, *Reason for test* and *METS*. All the features used in the explanation except *METS* are positively contributed to the prediction of low risk of hypertension (probability = 0.96). Figure 36 shows Shapley Values explanation for instance 14 based on the features of *Resting Systolic Blood Pressure*, *Age*, *METS*, *Hx Hyperlipidemia*, and *Resting Diastolic Blood Pressure*. All the features contributed toward increasing the probability of low risk of hypertension.

In the following, we present sample instances of *False Negative* predictions from *Group 5*. The instances are selected based on the patient’s age: one instance is close to the maximum age, one instance is close to the minimum age and one instance close to average age.

#### Instance 15 (False Negative Prediction of Low Risk - Group 5 - Close to Maximum Age)

The description of this instance is as follows: *Age = 79.6, METS = 7, Resting Systolic Blood Pressure = 120, Peak Diastolic Blood Pressure = 70, Resting Diastolic Blood Pressure = 64, HX Coronary Artery Disease = 0, Reason for test = Chest Pain,HX Diabetes = false, Percentage HR achieved = 0.96, Race = white, Hx Hyperlipidemia = true, Aspirin Use = false, Hypertension Response = true.*

**Fig. 37:**
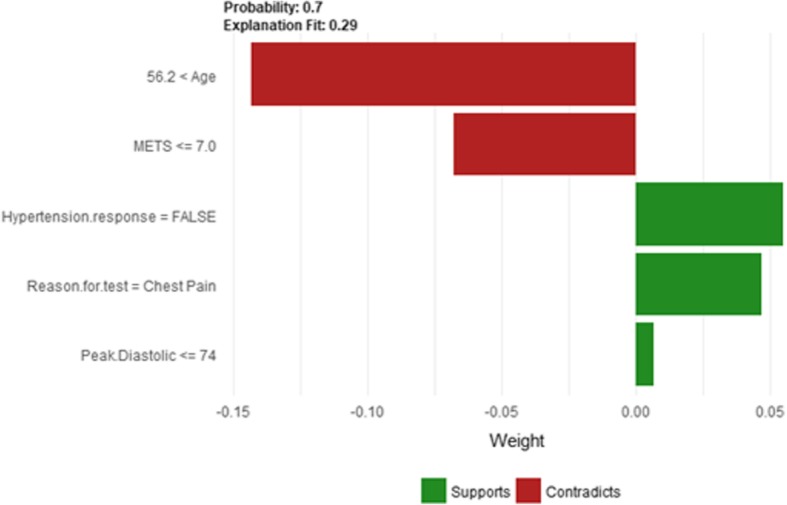
LIME explanation of Instance 15 as False Negative Prediction of Low Risk - Group 5 - Close to Maximum Age

**Fig. 38:**
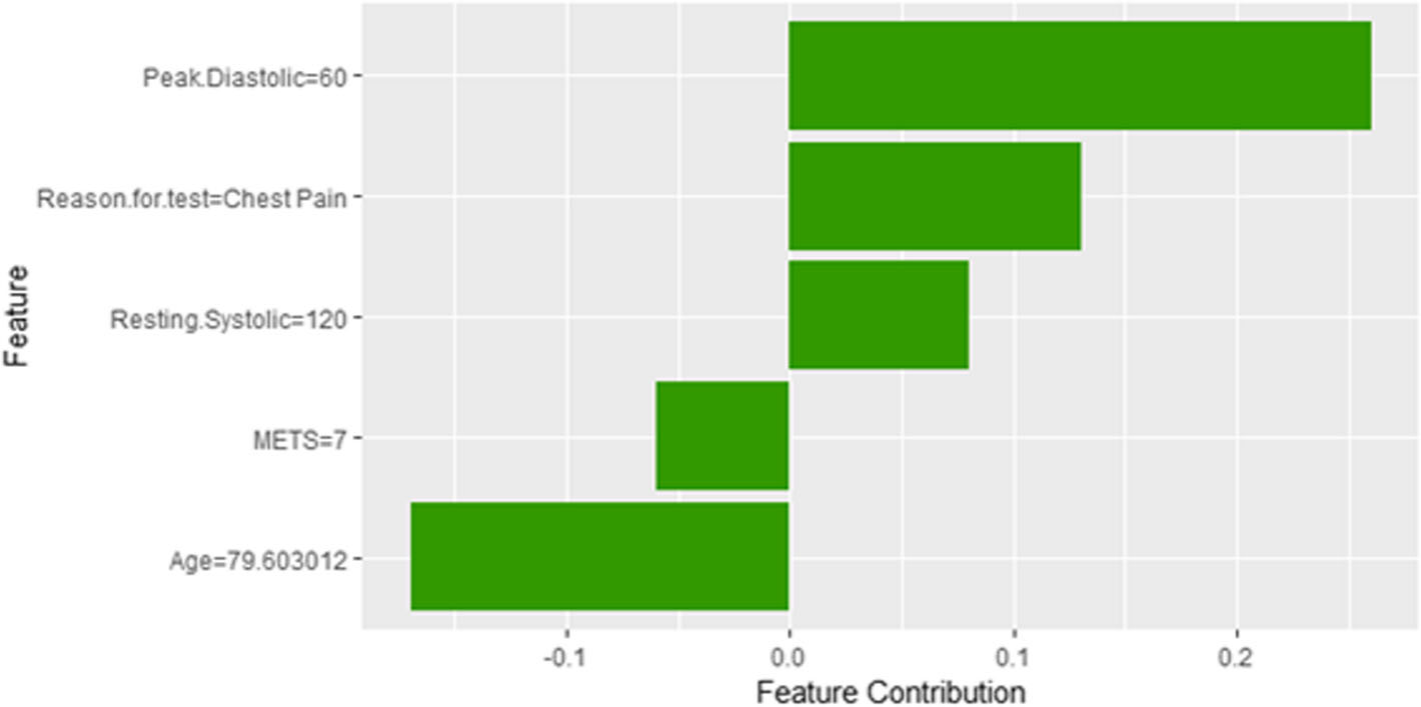
Shapley explanation of Instance 15 as False Negative Prediction of Low Risk - Group 5 - Close to Maximum Age

Figure 37 shows the explanation of instance 15 based on *Age*, *METS*, *Hypertension Response*, *Reason for test* and *Peak Diastolic Blood Pressure*. All the features used in the explanation except *Age* and *METS* are contributed positively to the prediction of low risk of hypertension with probability equals to 0.7. Shapley Values explanation for instance 15, shown in Fig. 38, is based on the same five features used by LIME except for *Hypertension Response* is replaced by Resting *Systolic Blood Pressure*. *Peak Diastolic Blood Pressure* and *Age* are the most contributing features toward increasing and decreasing the probability of low risk of hypertension respectively.

#### Instance 16 (False Negative Prediction of Low Risk - Group 5 - Close to Minimum Age)

The description of this instance is as follows: *Age = 22.78, METS = 12.9, Resting Systolic Blood Pressure = 112, Peak Diastolic Blood Pressure = 64, Resting Diastolic Blood Pressure = 68, HX Coronary Artery Disease = false, Reason for test = Dizzy, HX Diabetes = false, Percentage HR achieved = 1.01, Race = white, Hx Hyperlipidemia = true, Aspirin Use = false, Hypertension Response = false*.

**Fig. 39:**
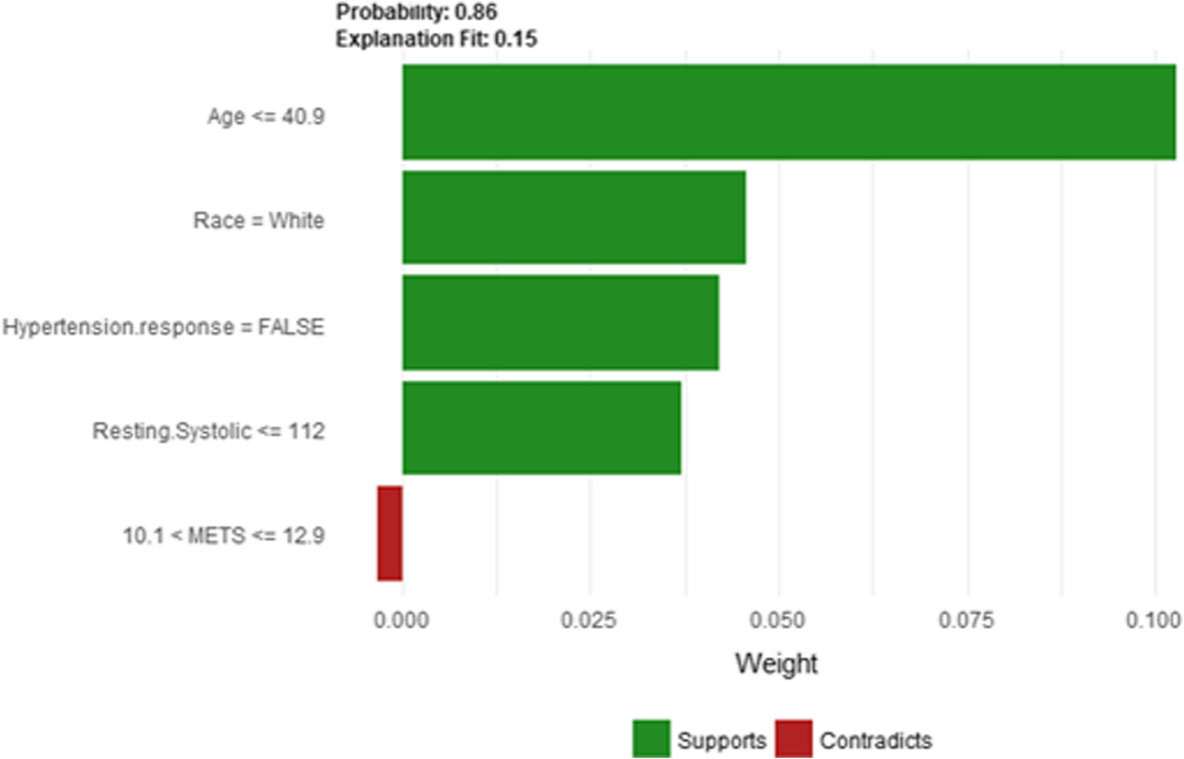
LIME explanation of Instance 16 as False Negative Prediction of Low Risk - Group 5 - Close to Minimum Age

**Fig. 40:**
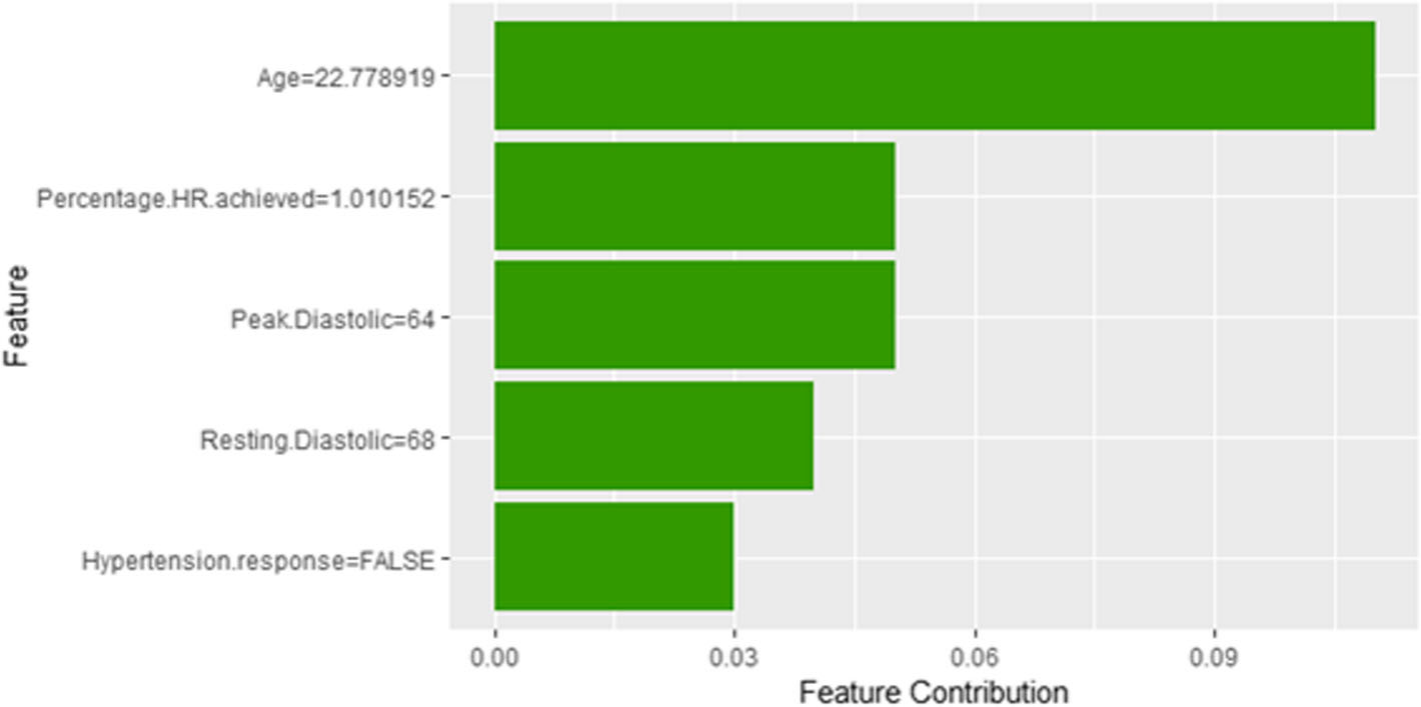
Shapley explanation of Instance 16 as False Negative Prediction of Low Risk - Group 5 - Close to Minimum Age

Figure 39 shows LIME explanation of instance 16 based on Age, Race, Hypertension Response, Resting Systolic Blood Pressure and METS. All the features used in the explanation except METS contributed positively to the prediction of low risk of hypertension with a strong probability of 0.86. Figure 40 shows Shapley Values explanation of instance 16 based on features Age, Percentage HR achieved, Peak Diastolic Blood Pressure, Resting Diastolic Blood Pressure, and Hypertension Response. All the features used in the explanation contributed toward increasing the probability of low risk of hypertension.

#### Instance 17 (False Negative Prediction of Low Risk - Group 5 - Close to Average Age)

The description of this instance is as follows*: Age = 48.78, METS = 10.1, Resting Systolic Blood Pressure = 110, Peak Diastolic Blood Pressure = 70, Resting Diastolic Blood Pressure = 70, HX Coronary Artery Disease = false, Reason for test = Rule out Ischemia,HX Diabetes = 0, Percentage HR achieved = 0.92, Race = black, Hx Hyperlipidemia = false, Aspirin Use = false, Hypertension Response = false*.

**Fig. 41:**
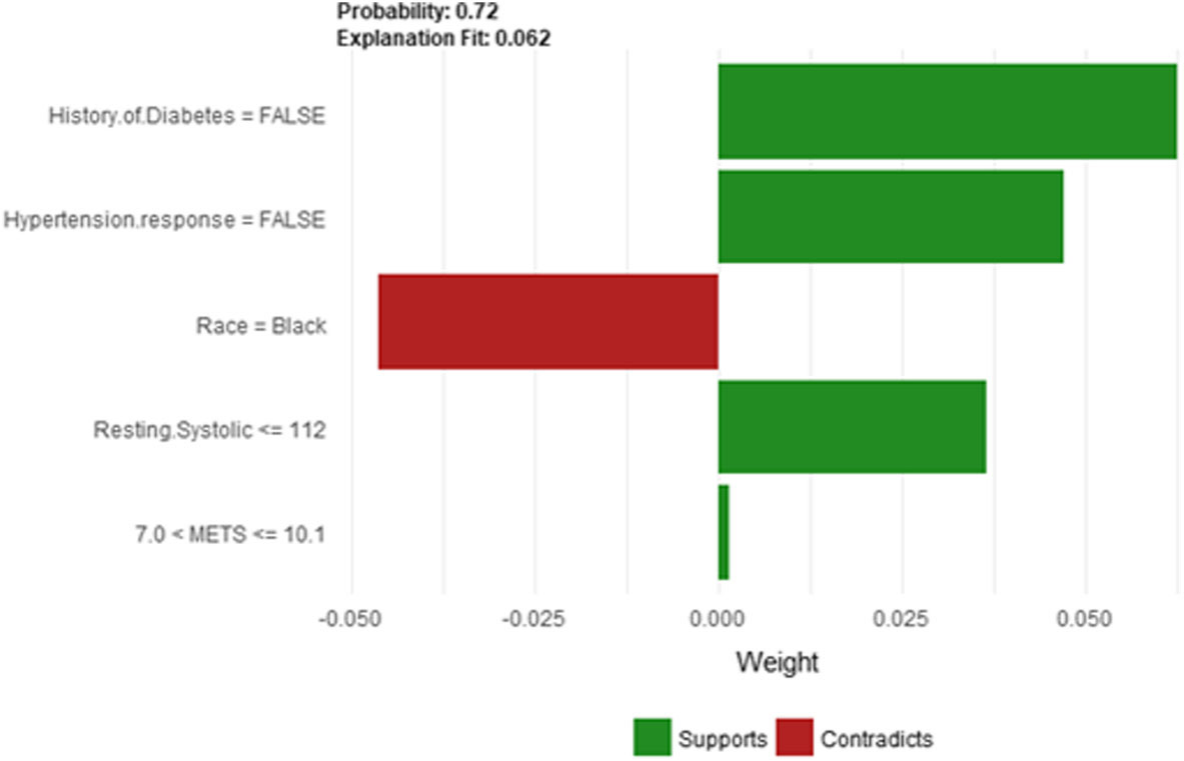
LIME explanation of Instance 17 as False Negative Prediction of High Risk - Group 5 - Close to average age

**Fig. 42:**
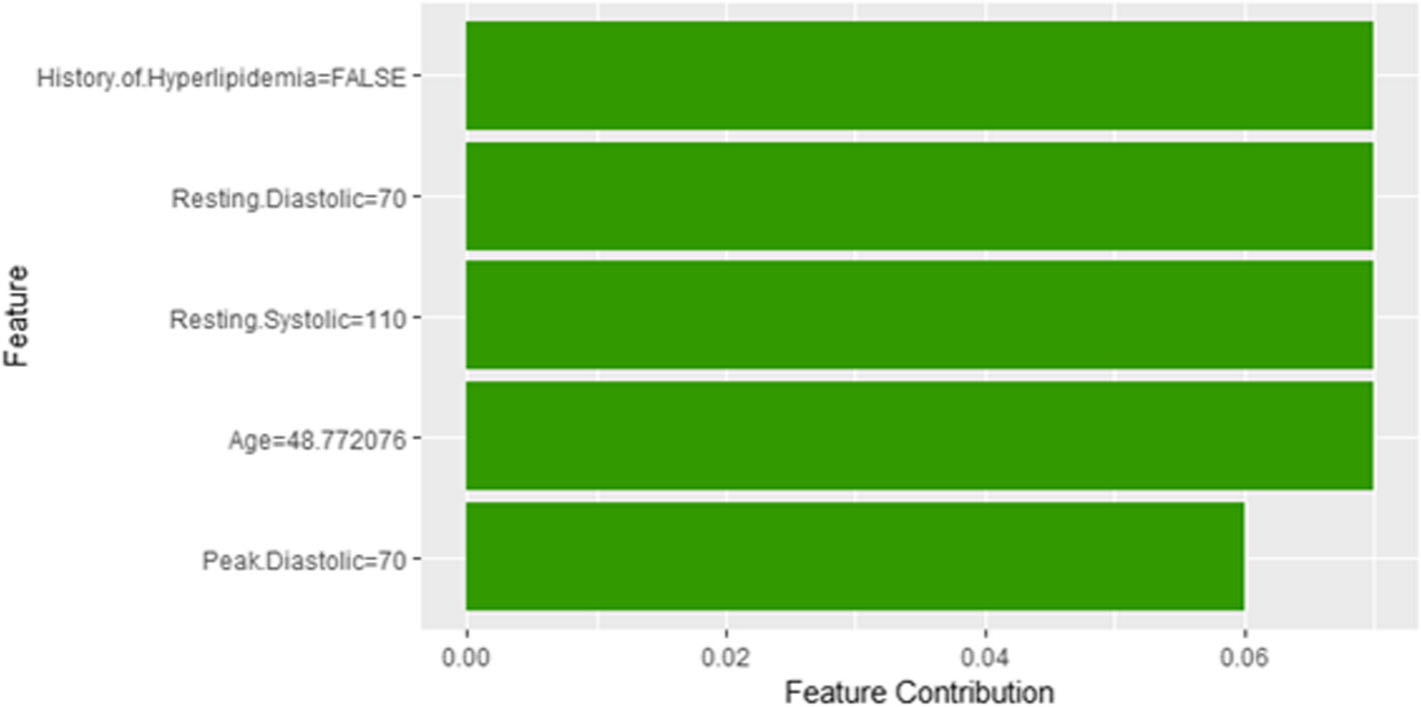
Shapley explanation of Instance 17 as False Negative Prediction of High Risk - Group 5 - Close to average age

Figure 41 shows the explanation of instance 17 based on HX Diabetes, Hypertension, Response, Race, Resting Systolic Blood Pressure and METS. All the features used in the explanation except being black are contributed to the prediction of low risk of hypertension with a probability of 0.72. Figure 42 shows Shapley Values explanation of instance 17 which is based on Hx Hyperlipidemia, Resting Diastolic Blood Pressure, Resting Systolic Blood Pressure, Age and Peak Diastolic Blood Pressure. All the features contributed toward increasing the probability of low risk of hypertension.

In the following, we present sample instances of *False Negative* predictions from *Group 6*. The instances are selected based on the patient’s age: one instance is close to the maximum age, one instance is close to the minimum age and one instance close to average age.

#### Instance 18 (False Negative Prediction of Low Risk - Group 6 - Close to Maximum Age)

The description of this instance is as follows: *Age* = 78.2, *METS* = 7, *Resting Systolic Blood Pressure* = 110, *Peak Diastolic Blood Pressure* = 84, *Resting Diastolic Blood Pressure* = 72, *HX Coronary Artery Disease* = false, *Reason for test* = chest pain, *HX Diabetes* = false, *Percentage HR achieved* = 0.96, *Race* = white, *Hx Hyperlipidemia* = false, *Aspirin Use* = false, *Hypertension Response* = false.

**Fig. 43:**
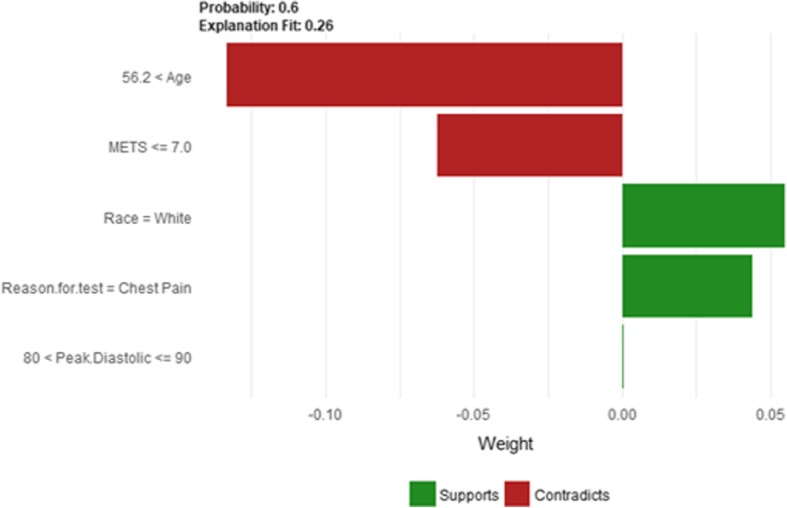
LIME explanation of Instance 18 as False Negative Prediction of Low Risk - Group 3 - Close to Maximum Age

**Fig. 44:**
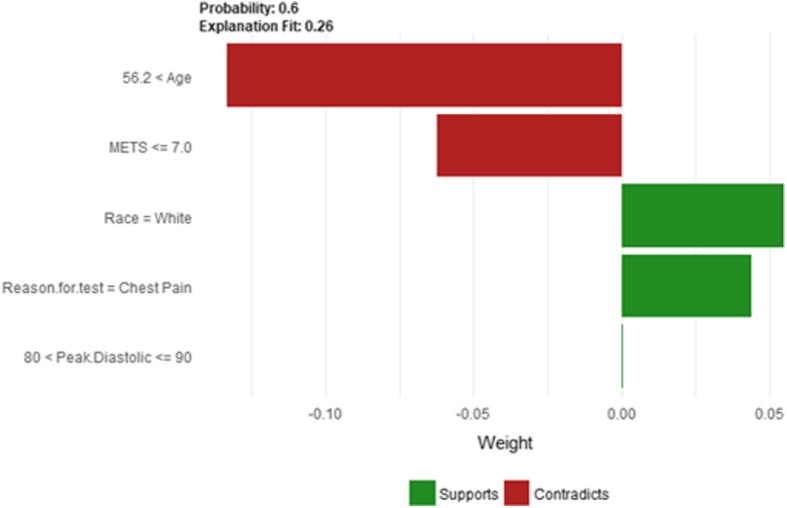
Shapley explanation of Instance 18 as False Negative Prediction of Low Risk - Group 3 - Close to Maximum Age

Figure 43 shows LIME explanation of instance 18 based on *Age*, *METS*, *Race*, *Reason for test*, and *Peak Diastolic Blood Pressure*. *Race* and *Reason for test* contributed positively to the prediction of low risk of hypertension with a weak probability of 0.6. Figure 44 shows Shapley Values explanation of instance 18 which is based on *Resting Systolic Blood Pressure*, *Resting Diastolic Blood Pressure*, *Reason for test*, and *Peak Diastolic Blood Pressure*, *Age*. All the features except *Age* contributed toward increasing the probability of low risk of hypertension.

#### Instance 19 (False Negative Prediction of Low Risk - Group 6 - Close to Minimum Age)

The description of this instance is as follows: *Age = 27.8, METS = 10.1, Resting Systolic Blood Pressure = 112, Peak Diastolic Blood Pressure = 110, Resting Diastolic Blood Pressure = 80, HX Coronary Artery Disease = false, Reason for test = shortness of breath, HX Diabetes = false, Percentage HR achieved = 0.86, Race = white, Hx Hyperlipidemia = false, Aspirin Use = false, Hypertension Response = false*.

**Fig. 45:**
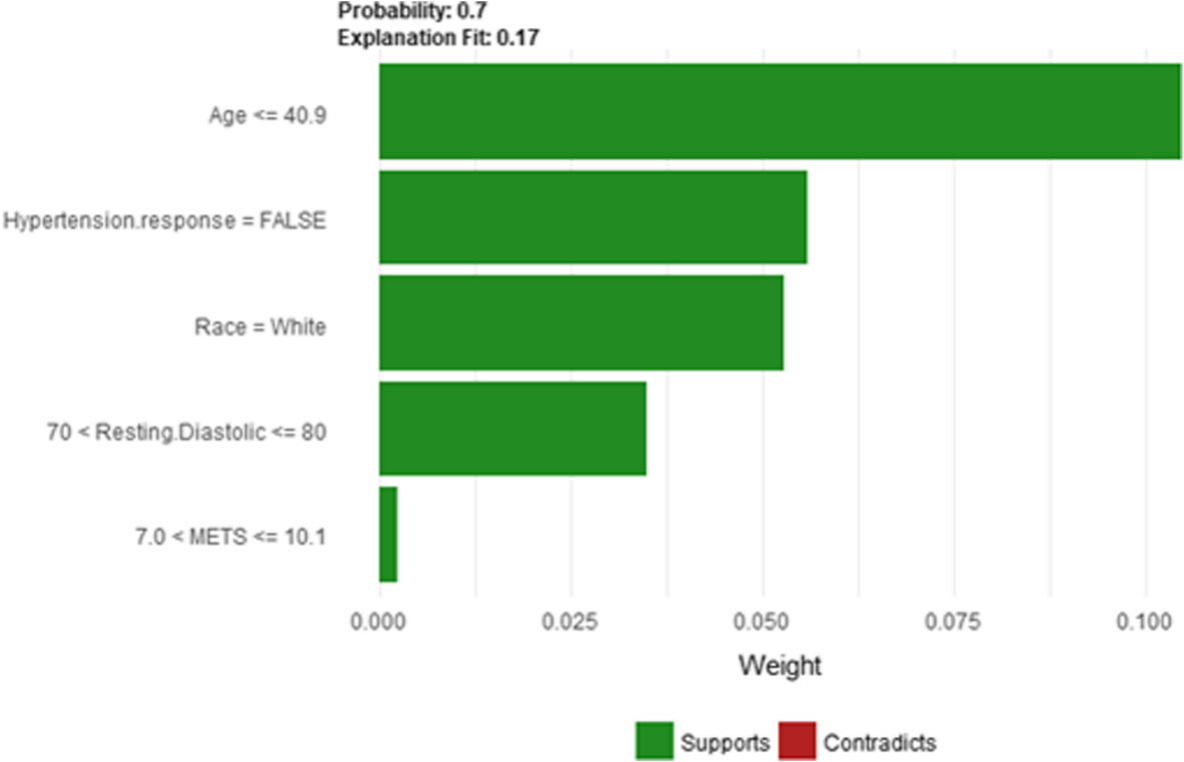
LIME explanation of Instance 19 as False Negative Prediction of Low Risk - Group 3 - Close to Minimum Age

**Fig. 46:**
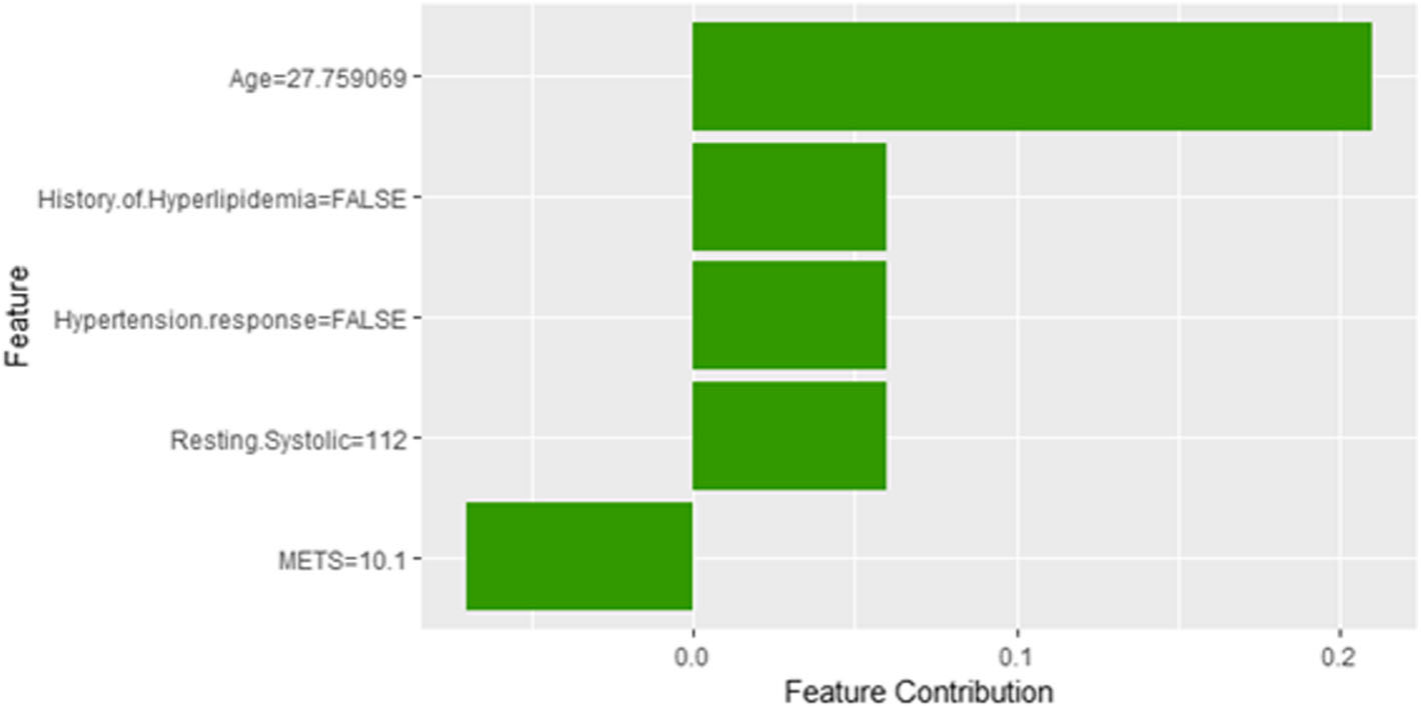
Shapley explanation of Instance 19 as False Negative Prediction of Low Risk - Group 3 - Close to Minimum Age

Figure 45 shows the explanation of instance 19 based on *Age*, *Hypertension Response*, *Race*, *Resting Diastolic Blood Pressure* and *METS* and. All the features used in the explanation contributed positively to the prediction of low risk of hypertension with a probability of 0.7. Figure 46 shows the Shapley Values explanation of instance 19 which is based on *Age*, *Hx Hyperlipidemia*, *Hypertension Response*, *Resting Systolic Blood Pressure*, and *METS*. All the features except *METS* contributed toward increasing the probability of low risk of hypertension.

#### Instance 20 (False Negative Prediction of Low Risk - Group 6 - Close to Average Age)

The description of this instance is as follows: *Age = 48.5, METS = 5, Resting Systolic Blood Pressure = 110, Peak Diastolic Blood Pressure = 88, Resting Diastolic Blood Pressure = 78, HX Coronary Artery Disease = false, Reason for test = shortness of breath, HX Diabetes = false, Percentage HR achieved = 0.9, Race = white, Hx Hyperlipidemia = false, Aspirin Use = false, Hypertension Response = false*.

**Fig. 47:**
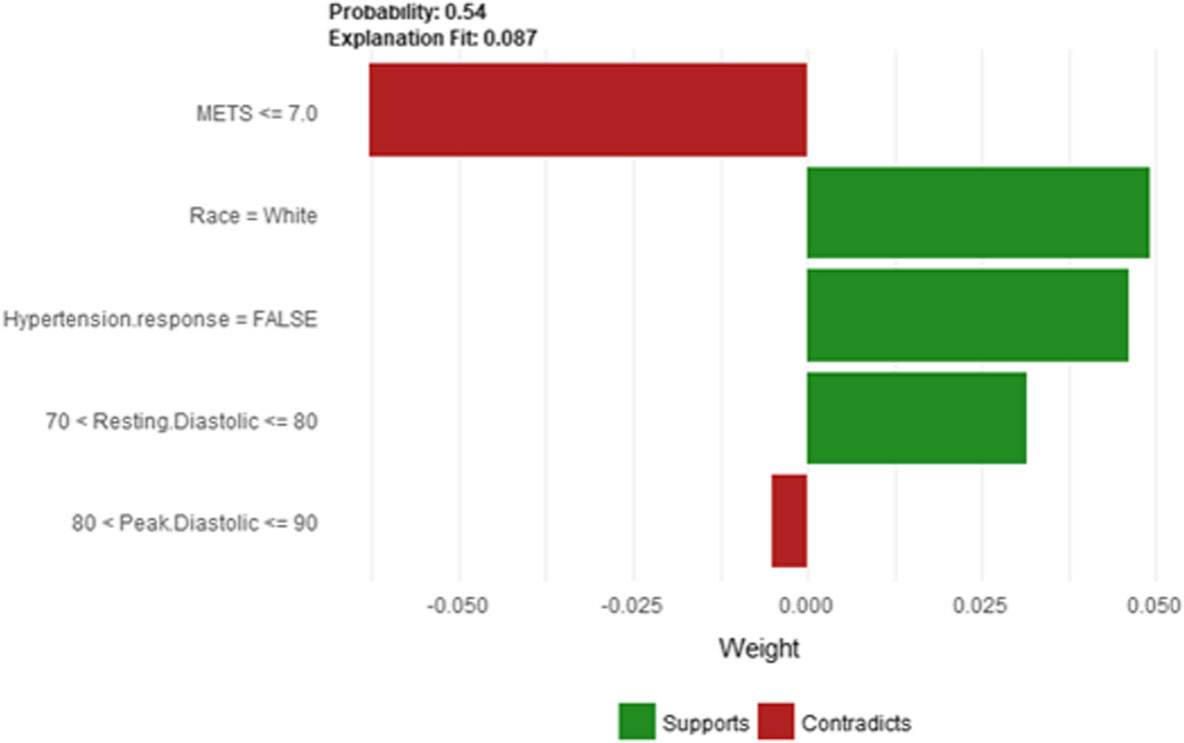
LIME explanation of Instance 20 as False Negative Prediction of Low Risk - Group 3 - Close to Average Age

**Fig. 48:**
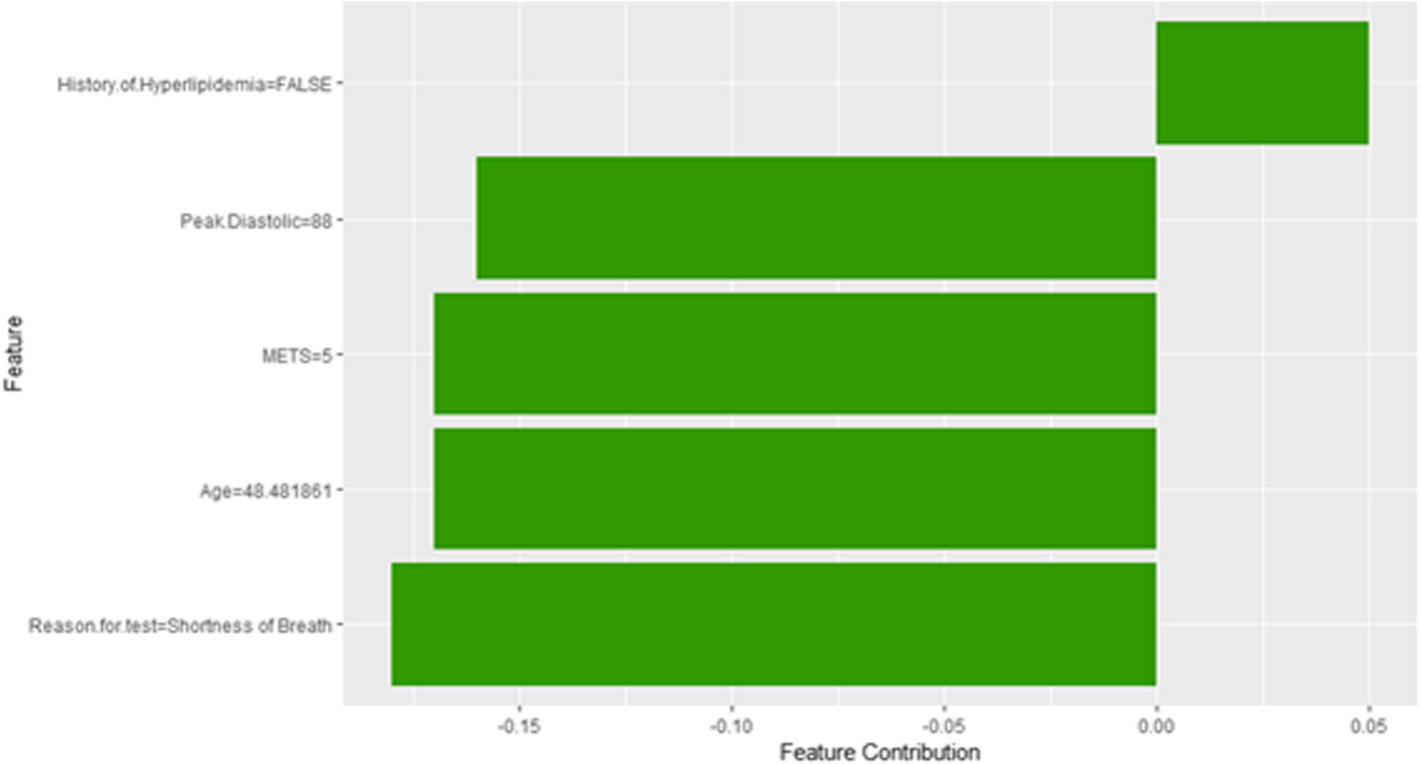
Shapley explanation of Instance 20 as False Negative Prediction of Low Risk - Group 3 - Close to Average Age

Figure 47 shows LIME explanation of instance 20 based on *METS*, *Race*, *Hypertension Response*, *Resting* Diastolic *Blood Pressure* and *Peak Diastolic Blood Pressure*. All the features used in the explanation except *METS* and *Peak Diastolic Blood Pressure* contributed to the prediction of low risk of hypertension with a weak probability of 0.54. Figure 48 shows the Shapley Values explanation of instance 20 based on *Hx Hyperlipidemia*, *Peak Diastolic Blood Pressure*, *METS*, *Age*, and *Reason for test*. All the features used in the explanation except *Hx Hyperlipidemia* contributed toward decreasing the probability of low risk of hypertension.

## Discussion

In general, the global interpretability techniques have the advantage that it can generalize over the entire population while local interpretability techniques give explanations at the level of instances. Both methods may be equally valid depending on the application need. For example, a healthcare application such as predicting the progression of risk of hypertension may require global understanding for the main risk factors for developing hypertension. In this case, local explainers may not be suitable. One way to meet the application goal is to use the global explanation methods. Another way to meet the application requirements using local explainers is to get local explanations and then aggregate them to generate global level explanations. Such technique is computationally expensive.

One of the main advantages of LIME is that its explanation is based on the local regression model, which allow physicians to make statements about changes in explanations for changes in the features of the patient to be explained, for example, “what would the probability of hypertension if the patients after five years?”. One of the main limitations of LIME is the instability of the explanations. Patients with very close characteristics may have very different explanations. Even for a single patient, if you get the explanation twice, you may get two different explanations. Another limitation is the perturbed data points that act as the training data for the interpretable model are sampled from Gaussian distribution that ignores the correlation between features. This may lead to poor selection of data points that result in poor explanation. LIME assumes a strong assumption that the local model fitted on the perturbed data is linear, however, there is no clear theory about the validity of the assumption.

One of the main advantages that distinguish Shapley value explanation from LIME is that the difference between the average prediction and the prediction of the instance to be explained is fairly distributed among the feature values of the instance to be explained. In other words, Shapley, value explanation. On the other side, Shapley value explanation is computationally expensive. Another disadvantage is that we need to access the training examples used in training the model to be explained unlike LIME.

Many methods have been proposed to make complex machine learning model interpretable, however, these methods have been evaluated individually on small datasets [60]. To the best of our knowledge, this is the first study that applies and demonstrates the utility of various model-agnostic explanation techniques of machine learning models analyzing the outcomes of prediction model for the individuals at risk of developing hypertension based on cardiorespiratory fitness data. This study is designed to take advantage of the unique and rich clinical research dataset consisting of 23,095 patients to explain the predictions of the best performing machine learning model for predicting individuals at risk of developing hypertension in an understandable manner for clinicians. The results show that different interpretability techniques can shed light on different insights on the model behavior where global interpretations can enable clinicians to understand the entire conditional distribution modeled by the trained response function. In contrast, local interpretations promote the understanding of small parts of the conditional distribution for specific instances. In practice, both methods can be equally valid depending on the application need. Both methods are effective methods for assisting clinicians on the medical decision process, however, the clinicians will always remain to hold the final say on accepting or rejecting the outcome of the machine learning models and their explanations based on their domain expertise.

## Threats to validity

### Extenral validity

A main limitation of this study is that the predictors of the models, the predictions of the models fot the new instances and the explanations of the interpretability techniques are all based on the charachteritsics and used predictors of the cohort of this study.

### Construct validity

This study has been mainly focusing on two local interpretability techniques, namely, LIME and Shapley Value Explanations. The inclusion of additional local interpretability techniques may lead to different explanations and additional insights.

### Conclusion Validity

Due to the nature of this study and the unlimited availability of similar comparable cohorts. Generalizing the findings and explanations of this study would require the inclusion of multiple datasets representing multiple cohorts.

## Conclusion

Explaining the predictions of black-box machine learning models have become a crucial issue which is gaining increasing momentum. In particular, achieving optimal performance of the machine learning models have not become the only focus of data scientists, instead, there is growing attention on the need for explaining the predictions of black-box models on both global and local levels. Several explanations that have been produced by various methods in this study reflect the significant role of these techniques in assisting the clinical staff in the decision-making process. For example, the LIME technique can allow physicians to make statements about changes in explanations for changes in the features of the patient to be explained. However, the LIME technique suffers from the instability of the explanations. Meanwhile, the Shapley value explanation technique has shown the ability to demonstrate that the difference between the average prediction and the prediction of the instance to be explained is fairly distributed among the feature values of the instance to be explained. On the other hand, Shapley value explanation is computationally expensive and needs to access the training data, unlike LIME. Finally, we believe that this study is an important step on improving the understanding and trust of intelligible healthcare analytics through inducting a comprehensive set of explanations for the prediction of local and global levels. As a future work, there are various directions to extend and build up on this work. For example, generalizing the explanation by the inclusion of multiple datasets representing multiple cohort. In addition, incorporationg additional local interpretability techniques and studying their impact. Furthermore, investigating how the outcomes of the various explanation techniques can be effectively utilized to update and improve the accuracy of the prediction model and consequently the quality of the provided interpretations.

## Data Availability

The FIT project includes data from a single institution which was collected under IRB approval and did not utilize public funding or resources. Resources from Henry Ford Hospital were utilized in this project. The IRB approval clearly stated that the data will remain with the PI (Dr. Mouaz Al-Mallah - mouaz74@gmail.com) and the study investigators. We would like to note that there many ongoing analyses from the project. Data sharing will be only on a collaborative basis after the approval of the all the investigators who have invested time and effort on this project. This also has to be subject to IRB approval from Henry Ford Hospital and data sharing agreements.

## Abbreviations

CRF: Cardiorespiratory Fitness
LIME: Local interpretable model-agnostic explanations
ML: Machine Learning
RF: Random Forest
